# The rejuvenating influence of young plasma on aged intestine

**DOI:** 10.1111/jcmm.17926

**Published:** 2023-08-23

**Authors:** Taha Ceylani, Hikmet Taner Teker, Seda Keskin, Gizem Samgane, Eda Acikgoz, Rafig Gurbanov

**Affiliations:** ^1^ Department of Molecular Biology and Genetics Muş Alparslan University Muş Muş Turkey; ^2^ Department of Food Quality Control and Analysis Muş Alparslan University Muş Muş Turkey; ^3^ Department of Molecular Biology Ankara Medipol University Ankara Ankara Turkey; ^4^ Department of Histology and Embryology Van Yuzuncu Yil University Van Turkey; ^5^ Department Biotechnology Institute of Graduate Education, Bilecik Şeyh Edebali University Bilecik Bilecik Turkey; ^6^ Department of Bioengineering Bilecik Şeyh Edebali University Bilecik Bilecik Turkey; ^7^ Central Research Laboratory (BARUM) Bilecik Seyh Edebali University Bilecik Bilecik Turkey

**Keywords:** aging, cell density, inflammation, intestinal tissue, IR spectroscopy, machine learning, plasma exchange, Sprague–Dawley rat

## Abstract

This study aims to investigate the effects of plasma exchange on the biomolecular profiles and histology of ileum and colon tissues in young and aged Sprague–Dawley male rats. Fourier transform infrared (FTIR) spectroscopy, linear discriminant analysis and support vector machine (SVM) techniques were employed to analyse the lipid, protein, and nucleic acid indices in young and aged rats. Following the application of young plasma, aged rats demonstrated biomolecular profiles similar to those of their younger counterparts. Histopathological and immunohistochemical assessments showed that young plasma had a protective effect on the intestinal tissues of aged rats, increasing cell density and reducing inflammation. Additionally, the expression levels of key inflammatory mediators tumour necrosis factor‐alpha and cyclooxygenase‐2 significantly decreased after young plasma administration. These findings underscore the therapeutic potential of young plasma for mitigating age‐related changes and inflammation in the intestinal tract. They highlight the critical role of plasma composition in the ageing process and suggest the need for further research to explore how different regions of the intestines respond to plasma exchange. Such understanding could facilitate the development of innovative therapies targeting the gastrointestinal system, enhancing overall health during ageing.

## 1 INTRODUCTION

The exploration of strategies to reverse, halt or delay the effects of ageing is a critical area of research. Studies conducted on various organisms have revealed that ageing is influenced by factors such as genomic instability, telomere attrition, epigenetic alterations, dysregulated nutrient sensing, impaired mitochondrial function, cellular senescence, decline in stem cell potential and disrupted intercellular signalling.^1^ Promisingly, investigations have indicated that young blood plasma may hold therapeutic potential in mitigating these age‐related damages. Heterochronic parabiosis studies, which involve the sharing of circulatory systems between young and aged animals, have demonstrated rejuvenating effects in various tissues, including muscle and liver,^2^ spinal cord,^3^ heart^4^ and pancreatic beta cells in aged rats.^5^ Young plasma components have been shown to enhance hippocampal synaptic activity and improve cognitive functions.^6^ Moreover, the administration of young plasma has been found to induce liver regeneration and restore age‐related liver functions in aged animals through the modulation of autophagy.^7^ Furthermore, young plasma has been found to alleviate Alzheimer's disease‐related brain pathologies and improve cognitive impairments in animal models.^8^ Recent investigations have also revealed that young plasma transfer not only increases sperm concentrations but also restores epigenetic patterns within the testes of aged animals.^9^ These findings underscore the potential therapeutic applications of young plasma in addressing age‐related changes and rejuvenating tissues in various physiological systems.

Infrared (IR) spectroscopy, a powerful tool for biological analyses, uses its multiprocessing abilities to gather extensive data rapidly and non‐invasively. It captures the vibrations of molecules to form spectral bands in the mid‐IR region^10^, ^11^ Combined with machine learning and other statistic data processing methods, the technique has been shown to be a promising tool in the diagnosis of diverse diseases.^12^, ^13^, ^14^

This study aims to investigate the therapeutic potential of young plasma in intestinal ageing. We hypothesize that young plasma can rejuvenate the biomolecular profile and structural integrity of aged rat ileum and colon tissues. We utilize advanced techniques like Fourier transform infrared (FTIR) spectroscopy, machine learning, histopathology, and immunohistochemistry to comprehensively analyse these potential changes. Additionally, we aim to compare the responses of ileum and colon tissues in young and aged rats to plasma exchange, providing insights into the differential impacts of ageing on these intestinal regions. Our findings will contribute to the development of novel therapeutic strategies for promoting gut health and longevity during the ageing process.

## 2 MATERIAL METHODS

### 2.1 Animal studies

Male Sprague–Dawley rat species was used as a model organism in the study. Aged rats (24 months *n* = 7) were treated with pooled plasma (0.5 mL per day for 30 days, intravenously into the tail vein) collected from young (5 weeks, n = 51) rats and young rats (5 weeks, *n* = 7) were treated with pooled plasma (0.25 mL per day for 30 days, intravenously into the tail vein) collected from aged (24 months *n* = 16) rats. After the plasma application, the rats in the experimental and control groups were slightly stunned with ether and sacrificed. The ileum and colon tissues were taken and shocked on dry ice, then labelled and left in the −80 cabinet until it was time to be studied. All animals were housed under standard animal care conditions and had free access to food and water. Our study was carried out with the approval of the Ethics Committee (approval number: 2021/03) from the Saki Yenilli Experimental Animal Production and Practice Laboratory.^6^, ^15^

### 2.2 Plasma collection

Pooled rat plasma was collected by terminal cardiac puncture at the time of euthanasia. Plasma was prepared from blood collected with EDTA, followed by centrifugation at 1000*g*. For plasma denaturation, plasma was heated for 2–3 min at 95°C, followed by a short spin at 1000 g. All plasma aliquots were stored at −80°C until use. Before administration, plasma was dialysed using 3.5‐kDa D‐tube dialysers (EMD Millipore) in PBS to remove EDTA.^6^, ^15^

### 2.3 Analysis of samples by attenuated total reflectance Fourier transform infrared spectroscopy

Ileum and colon samples of all animals were compressed on the Zn/Se crystal of the ATR unit (PerkinElmer) without any pretreatment and examined with an ATR‐FTIR spectrometer (PerkinElmer) at a resolution of 4 cm^−1^ and a scan number of 32. The spectra were obtained with the Spectrum One (PerkinElmer) software in the wavelength range of 4000–650 cm^−1^.^16^

### 2.4 Prediction studies with a machine learning approach based on big spectral data

Linear discriminant analysis (LDA), a machine learning approach, was applied to differentiate the experimental groups. Spectral data were used in pattern recognition analysis. Each sample spectrum was preprocessed on The Unscrambler® X 10.3 (CAMO Software AS) software with a baseline offset transformation in the 4000–650 cm^−1^ region for making the analyses as independent as possible from the FTIR spectrometers. Spectra processed in this way were first subjected to principal component analysis (PCA), an unsupervised pattern processing technique.^17^, ^18^ Spectra were passed from standard deviation normalization (mean centring normalization) and leverage or full‐cross random validation. Subsequently, the spectra were examined in lipid (3000–2700 cm^−1^), protein (1700–1500 cm^−1^), nucleic acid and polysaccharide (1200–650 cm^−1^) and full (4000–650 cm^−1^) regions by singular value decomposition algorithm.

LDA is a supervised classifier in which n‐dimensional feature samples are linearly transformed into an m‐dimensional space. PCA only uses sample spectra to determine the transformation, while LDA also uses class information in training samples leading to better classification. PCA data were utilized as LDA model inputs with The Unscrambler® X 10.3 (CAMO Software AS) multivariate analysis (MVA) software. The category variable column was included in a data matrix, and then all spectra of different sample categories were used to generate a training set. The linear method using the projections of the 7 PCA components was used for the prediction. Prior probabilities were calculated from the training set. The results are presented as a discrimination plot, prediction and confusion matrices.^17^, ^18^

The same spectral processings were also applied in support vector machine (SVM) modelling, using The Unscrambler® X 10.3 (CAMO Software AS) MVA software. All spectra of different sample categories were used to generate a training set. Classification (nu‐SVC) was chosen as the SVM type using a linear method as the Kernel type. Nu value was set to 0.5, weights as all 1.00. The seven cross‐validation segments were used to calculate training and cross‐validation accuracies. Finally, the generated training dataset was applied to all sample datasets to obtain an SVM classification model.^19^

### 2.5 Histopathological analysis

Biopsies were taken from the ileum and colon and then fixed in 10% neutral‐buffered formalin for a week at room temperature. Paraffin sections with a thickness of 4–5 μm were prepared using a rotary microtome (Leica Biosystems). The sections obtained were stained with haematoxylin and eosin stain (Haematoxylin: Cas No: 517‐28‐2; Eosin: Cas No: 17372‐87‐1, Merck, Germany) to visualize the general architecture of the rat ileum and colon. Random evaluation of approximately 10–15 areas per animal section was performed to observe histological alterations.^20^ Histopathological changes were evaluated under a light microscope by two blinded researchers.

### 2.6 Haematoxylin and eosin stain staining

After fixing, the tissues were dehydrated and embedded in paraffin at room temperature. Next, a continuous cross‐section with a thickness of 5 μm was made. These sections were then stained with haematoxylin and eosin dye. First, xylene and gradient ethanol were used for dewaxing. Next, the slices were treated with haematoxylin, 1% hydrochloric alcohol, tap water, and eosin. After staining, the sections were dehydrated in gradient ethanol and xylene, followed by mounting with entellan. Histopathologic changes of ileum and colon were observed under an optical light microscope (Nikon Eclipse Ni microscope, Nikon DS‐Fi2 camera; magnification ×100, ×200, and ×400).

### 2.7 Toluidin blue (TB) staining

The staining procedure for the identification of mast cells was the basic stain TB.^21^ All sections were stained with 0.1% TB stock solution as follows: 1 gr TB O (Sigma Aldrich) 100 mL 70% alcohol, were mixed to dissolve and then sodium chloride (1%) was added, pH is adjusted to 2.0–2.5 using glacial acetic acid. 5 μm sections were cut, dried and deparaffinized to distilled water, embedded in TB working solution for 3–10 min, washed in distilled water (3 times), dehydrated quickly through 95% and 2 times of 100% alcohol and finally 2 times were cleared in xylene. Mast cell granules, which are chromotropes, turn deep violet due to metachromasia.^22^ All the staining steps were conducted at room temperature.

### 2.8 Quantification of histopathological parameters

To evaluate lymphatic infiltration in the rat ileum and colon sections, a grayscale binarization approach with the lowest threshold level was employed to distinguish between purple‐stained and non‐stained areas. The area fractions (%) were quantified using Image J Fiji software (National Institutes of Health).

For quantification of Paneth cell density in the ileum, sections of all test and control subjects were stained with haematoxylin and eosin stain, and cells were counted in 30–40 crypts in representative microscopic fields (×400) (*n* = 7 per group).^23^

For mast cell distribution density, TB‐stained slides were evaluated under the light microscope (Nikon) with an integrated digital camera. To determine the distribution of mast cells in the slides stained with TB, Image J Fiji was used for mast cell density. Mast cells were counted at 40× objective magnification. The arithmetic average of these numbers was calculated by measuring the mast cells in all sections. Then, the average mast cells were determined as % area density. Mast cell density analyses were performed using Image J (Fiji) 1.54d package program (NIH). For TB staining, a similar grayscale binarization technique with the lowest threshold level was utilized to identify purple‐magenta stained areas, and the mast cell density area was quantified using Image J Fiji.^24^ Signal intensities from five images within each section were visually presented for all animals in the same group. Analysis of the microphotographs was performed using a light microscopy system comprising a Nikon Eclipse Ni microscope (Tokyo, Japan) with a camera attachment (Nikon DS‐Fi2), and imaging software (NIS Elements F 4.00.00, Nikon Soft Imaging Solution) at a magnification of 400×.

### 2.9 Immunohistochemical (IHC) analysis

IHC was performed using the previously described protocol for paraffin sections.^25^ After deparaffinization, endogenous peroxidase activity was inhibited by incubation with 3% hydrogen peroxide for 10 min and samples were washed with PBS. Heat‐induced antigen retrieval was performed by incubation in citrate buffer and the sections were incubated in a blocking solution for 10 min to prevent non‐specific binding. After the blocking step, all sections were incubated overnight with an anti‐tumour necrosis factor‐alpha (TNF‐α) (E‐AB‐52065, 1:150, Elabscience) and anti‐cyclooxygenase‐2 (anti‐COX‐2) primer antibodies (E‐AB‐17010, 1:150, Elabscience). The next day, biotinylated antibodies (TP‐125‐BN, Thermo Scientific) and streptavidin peroxidase (TS‐125‐HR) were applied to the sections. Then, a 3,3′‐Diaminobenzidine substrate kit (ab64238, Abcam) was performed for 3–5 min. Finally, the sections were counterstained with Mayer's haematoxylin and visualized using Nikon Eclipse Ni microscope with a Nikon DS‐Fi2 camera attachment. Images were imported into ImageJ (Fiji) for quantification of percent area of TNF‐α and COX‐2 expression analyses. TNF‐α and COX‐2 intensity (% Area) measurements in the ileum and colon were performed over 10 random areas of interest (3 different sections for each rat per group).^26^

### 2.10 Statistics

Statistical evaluations and graph plots of the results were made using GraphPad Prism 8.01 (GraphPad). The quantitative differences in various spectrochemical indices were statistically compared between Y**O**cnt (**O**ld control rats) and YOpls (**Y**oung plasma transferred **O**ld rats) as well as O**Y**cnt (**Y**oung control rats) and OYpls (**O**ld plasma transferred **Y**oung rats) groups to reveal the restoring and worsening effects of plasma administration on intestinal tissues. The data were analysed using an unpaired *t*‐test, and the significance levels were stated as *p* ≤ 0.05 *, *p* ≤ 0.01 **, *p* ≤ 0.001 ***, and *p* ≤ 0.0001 ****. Results are presented as mean ± SEM (standard error of the mean).

## 3 RESULTS

### 3.1 Plasma exchange affects the biomolecular profile of the ileum

**FIGURE 1:**
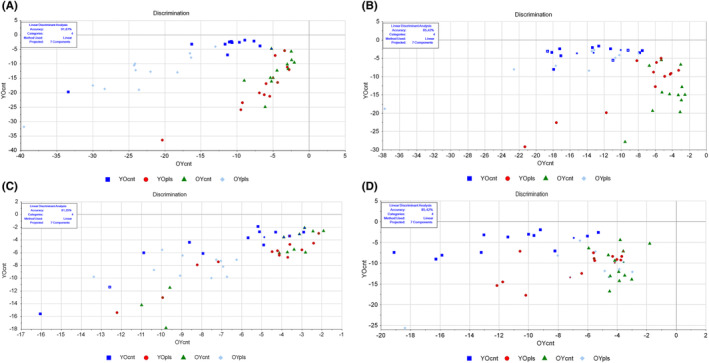
Linear discriminant analysis discrimination plots are provided for lipid (ranging from 3000 to 2700 cm^−1^) and protein (ranging from 1700 to 1500 cm^−1^) spectral regions. The plots are presented separately for ileum (A) and (B) and colon tissues (C) and (D). YOcnt (aged control rats), OYcnt (young control rats), YOpls (young plasma recipient aged rats), and OYpls (aged plasma recipient young rats).

Plasma exchange in rats had distinct effects on ileum tissues. Accuracies of ileum samples were 91.67% for lipids and 85.42% for proteins (Figure 1A,B). We observed a noteworthy trend when we examined the ileum samples of aged rats receiving young plasma (YOpls samples) and those of young control rats (OYcnt samples). The lipid and protein profiles of these two groups demonstrated striking similarities, and the same congruence was noted between young rats infused with aged plasma (OYpls samples) and the aged control rats (YOcnt samples). These results strongly suggest that the infusion of young plasma has a rejuvenating effect on the ileum lipids and proteins of aged rats. Conversely, the lipids and proteins of young rats exhibit signs of ageing when subjected to plasma from aged animals. This suggests that young plasma rejuvenates aged rat ileum while aged plasma accelerates ageing in young rat ileum (Figure 1A,B). LDA classification revealed accurate predictions and minimal confusion (Tables S1–S4). Whole biomolecules (lipids, proteins, nucleic acids, polysaccharides, etc.) and nucleic acids of the ileum showed a similar trend, excluding old plasma transferred to young rat samples (Figures S1 and S2). Samples were mostly accurately predicted (Tables S5–S8). The SVM method showed lower accuracy than LDA for whole ileum biomolecules (Table S9).

**FIGURE 2:**
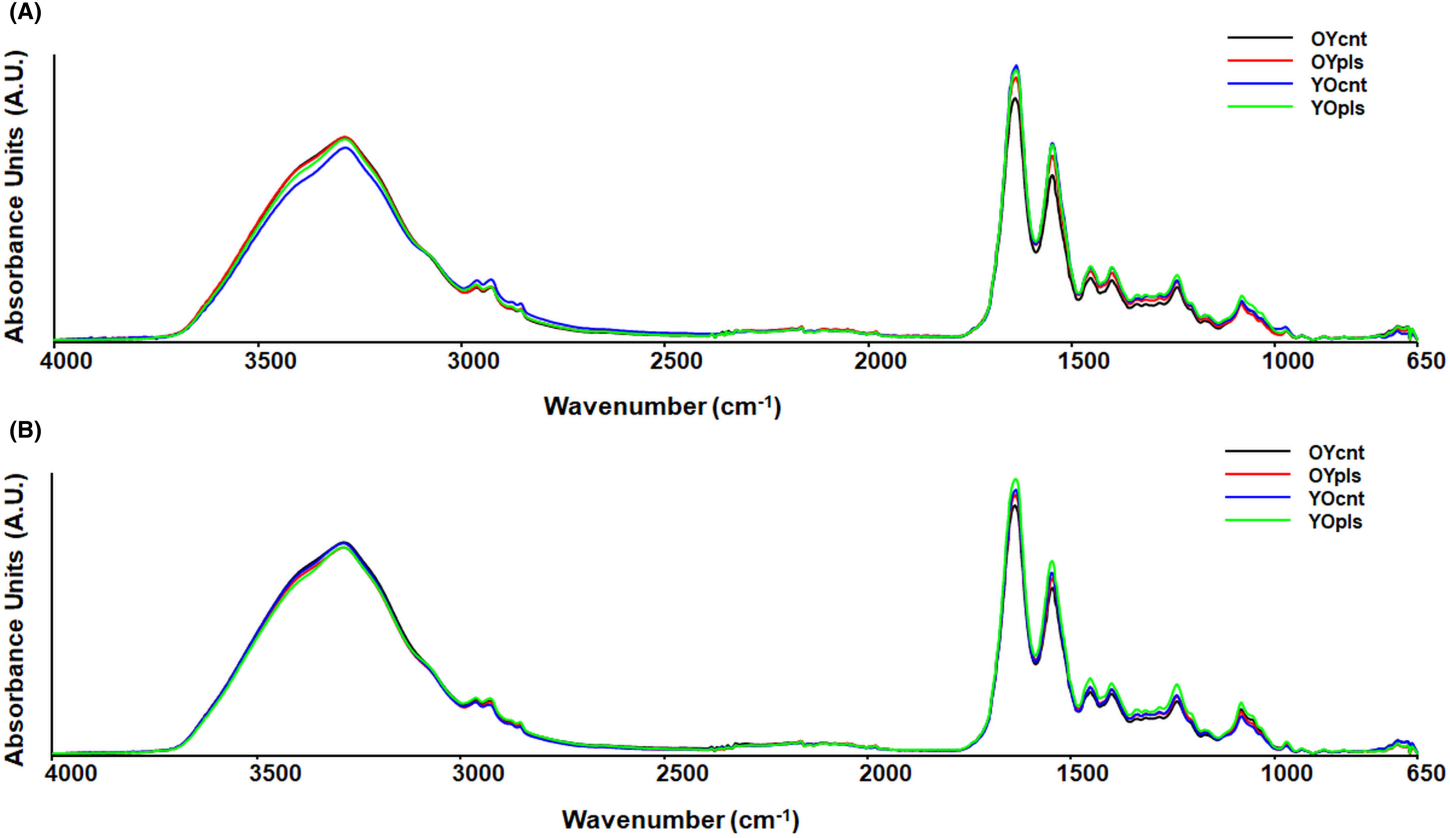
Baseline‐corrected average infrared spectra in the 4000–650 cm^−1^ spectral region for (A) ileum and (B) colon tissues. YOcnt (aged control rats), OYcnt (young control rats), YOpls (young plasma recipient aged rats), and OYpls (aged plasma recipient young rats).

**FIGURE 3:**
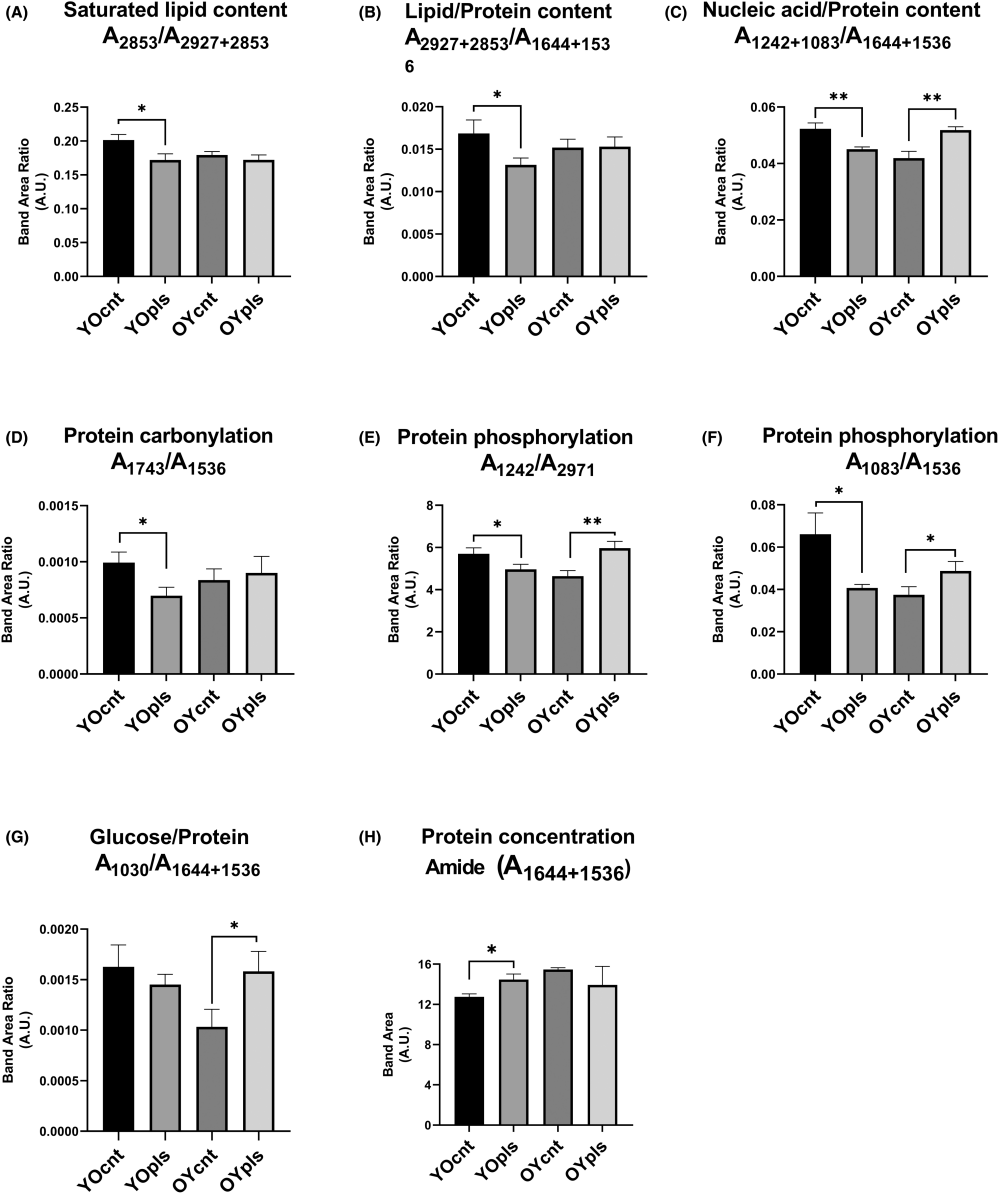
The quantitative changes in various spectrochemical parameters for ileum tissues. The indices for (A) saturated lipid content (A_2853_/A_2927+2853_), (B) lipid/protein content (A_2927+2853_/A_1644+1536_), (C) nucleic acid/protein content (A_1242+1083_/A_1644+1536_), (D) protein carbonylation (A_1743_/A_1536_), (E) and (F) protein phosphorylation (A_1242/_A_2971_; A_1083_/A_1536_), (G) glucose/protein (A_1030_/A_1644+1536_) and (H) amide protein concentrations (A_1644+1536_). YOcnt (aged control rats), OYcnt (young control rats), YOpls (young plasma recipient aged rats), and OYpls (aged plasma recipient young rats).

Beer's Lambert law was used to analyse the intensity and area of IR absorption bands, enabling the calculation of relative biomolecule concentrations. The study noted changes in the IR spectral bands, pointing to possible chemical modifications in lipids, proteins, and nucleic acids due to plasma exchange.^11^ The baseline‐corrected average IR spectra in the 4000–650 cm^−1^ spectral region for ileum and colon tissues are displayed in Figure 2. The measurement of quantitative changes in spectrochemical indices related to different biomolecules of ileum revealed a 14% and 21% decrease in order of saturated lipid (A_2853_/A_2927+2853_) and lipid/protein (A_2927+2853_/A_1644+1536_) contents in aged rats receiving young plasma (Figure 3A,B). A 13% reduction in the spectral index for nucleic acid/protein content (A_1242+1083_/A_1644+1536_) occurred after young plasma transfer in old rats (YOpls group). In comparison, this index was found to be higher by 23% after old plasma transferred to young rats (OYpls group) (Figure 3C). A 29% reduction of protein carbonylation index (A_1743_/A_1536_) occurred after young plasma transfer in aged rats (Figure 3D). Although the indices for protein phosphorylation (A_1242_/A_2971_ and A_1083_/A_1536_ ratios) decreased by 13%–38% after young plasma transfer to aged rats, these indices increased by 28%–30% in the ileum of young rats receiving aged plasma (Figure 3E,F). After young plasma transfer, there was no significant difference in the glucose/protein (A_1030_/A_1644+1536_) index in YOpls group, whereas aged plasma transfer increased this index by 53% in OYpls group (Figure 3G). A 14% increase in amide protein concentrations (A_1644+1536_) was measured in ileum tissues after young plasma transfer (Figure 3H).

### 3.2 Plasma exchange affects the biomolecular profile of the colon

Colon samples also presented notable findings. Discrimination accuracies were 81.25% for lipids and 85.42% for proteins (Figure 1C,D). Aged rats receiving young plasma (YOpls) and young rats receiving old plasma (OYpls) generally resembled their respective control groups. However, some YOpls and OYpls rats showed characteristics of both control groups, hinting at overlap in traits (Figure 1C,D; Figures S3 and S4). The results were further supported by confusion and prediction matrices of classes (Tables S10–S17). For whole biomolecules, SVM classified colon samples with 85.42% training and 54.16% cross‐validation accuracies. With seven out of 48 samples misclassified, SVM accuracy was deemed higher than LDA, which mispredicted 11 samples (Table S18).

**FIGURE 4:**
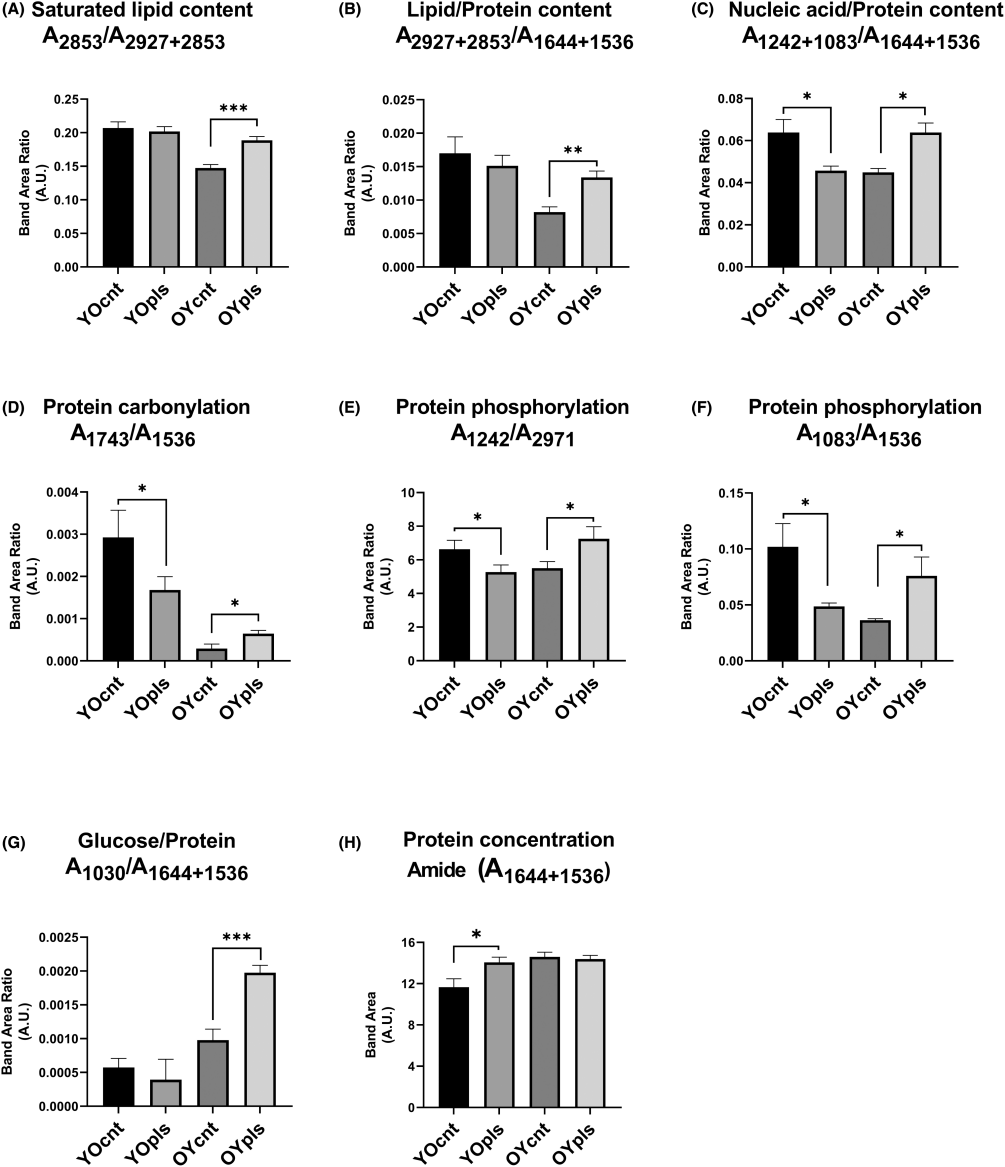
The quantitative changes in various spectrochemical parameters for colon tissues. The indices for (A) saturated lipid content (A_2853_/A_2927+2853_), (B) lipid/protein content (A_2927+2853_/A_1644+1536_), (C) nucleic acid/protein content (A_1242+1083_/A_1644+1536_), (D) protein carbonylation (A_1743_/A_1536_), (E) and (F) protein phosphorylation (A_1242/_A_2971_; A_1083_/A_1536_), (G) glucose/protein (A_1030_/A_1644+1536_) and (H) amide protein concentrations (A_1644+1536_). YOcnt (aged control rats), OYcnt (young control rats), YOpls (young plasma recipient aged rats), and OYpls (aged plasma recipient young rats).

IR spectra of colon samples also show the main differences. An index for saturated lipid content (A_2853_/A_2927+2853_) increased by 28% only after the aged plasma transfer to young rats (Figure 4A). In contrast to the ileum, there was a 63% increase in lipid/protein index (A_2927+2853_/A_1644+1536_) after the aged plasma transfer in the colon (Figure 4B). The changes (28% reduction in the YOpls group and 42% increment in the OYpls group) in nucleic acid/protein index (A_1242+1083_/A_1644+1536_) in colon tissues were similar to the ileum (Figure 4C). As in the ileum, carbonylation (A_1743_/A_1536_) and phosphorylation (A_1242_/A_2971_ and A_1083_/A_1536_) of proteins decreased in the order of 57% and 20%–52% in the colon of aged rats receiving young plasma transfer, while these indices increased in the order of 121% and 31%–109% after aged plasma transfer to young rats (Figure 4D,F). A 101% increase in glucose/protein index (A_1030_/A_1644+1536_) was depicted in colon tissues (Figure 4G). A 20% increase in amide protein concentrations (A_1644+1536_) was depicted in colon tissues after young plasma transfer (Figure 4H).

### 3.3 Histopathological alterations in the old and young plasma recipient rats' ileum and colon tissues

**FIGURE 5:**
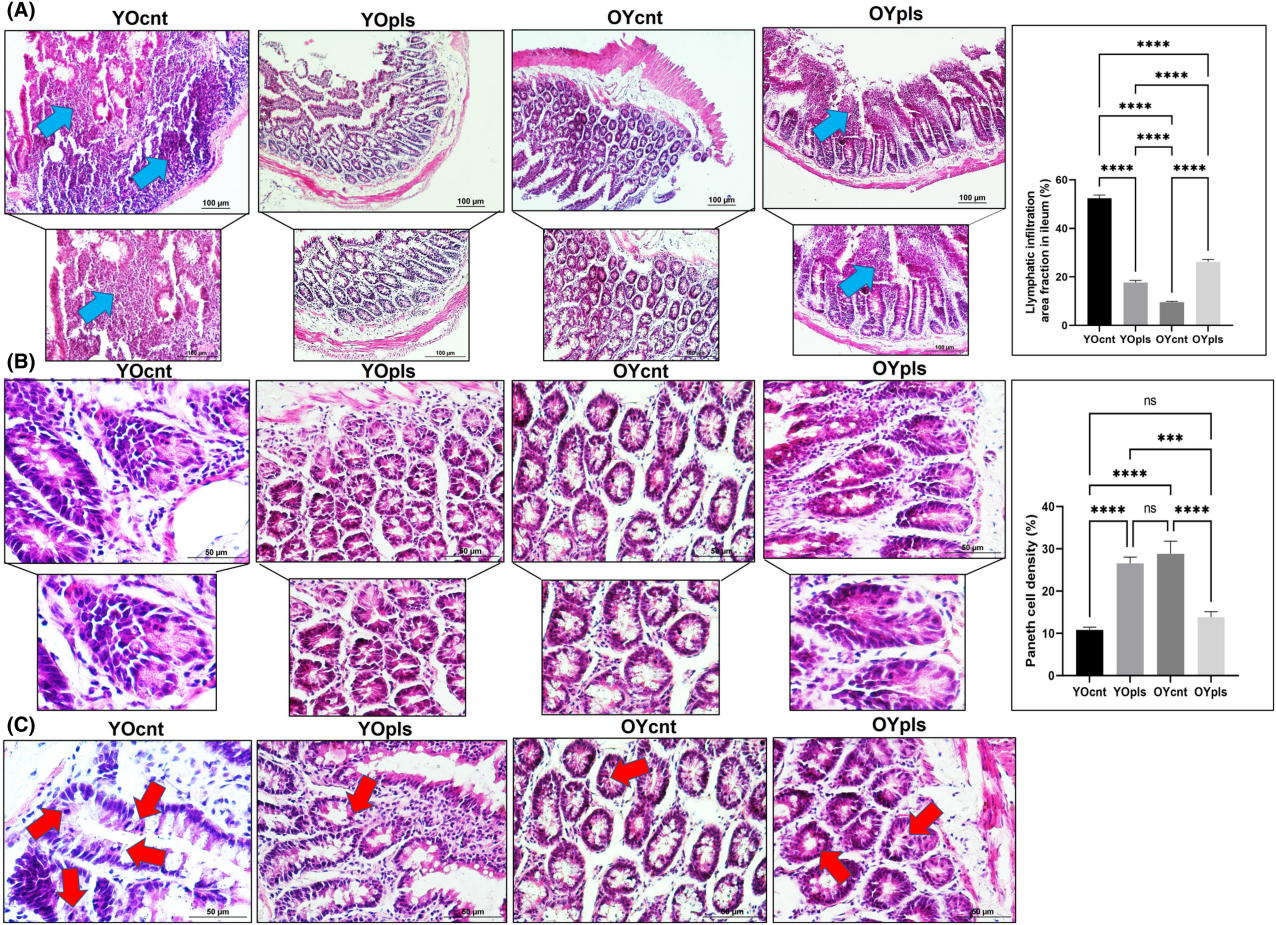
Representative images of haematoxylin and eosin stain staining and quantification of lymphatic infiltration area fraction (%) in all groups of ileum sections. Aged rats’ ileum show increased lymphatic infiltration, but young plasma received aged groups improved these histopathological alterations. The blue arrow shows lymphatic infiltrates. Areas in the haematoxylin and eosin stain‐stained microphotographs of all groups were magnified in the photos to which they belonged to the area of interest. Values are expressed as mean ± SEM; *n* = 7 rats in each group. *p* ≤ 0.0001**** (nonparametric Mann–Whitney *U*‐test). Scale bar = 100 μm. YOcnt (aged control rats), OYcnt (young control rats), YOpls (young plasma recipient aged rats), and OYpls (aged plasma recipient young rats).

**FIGURE 6:**
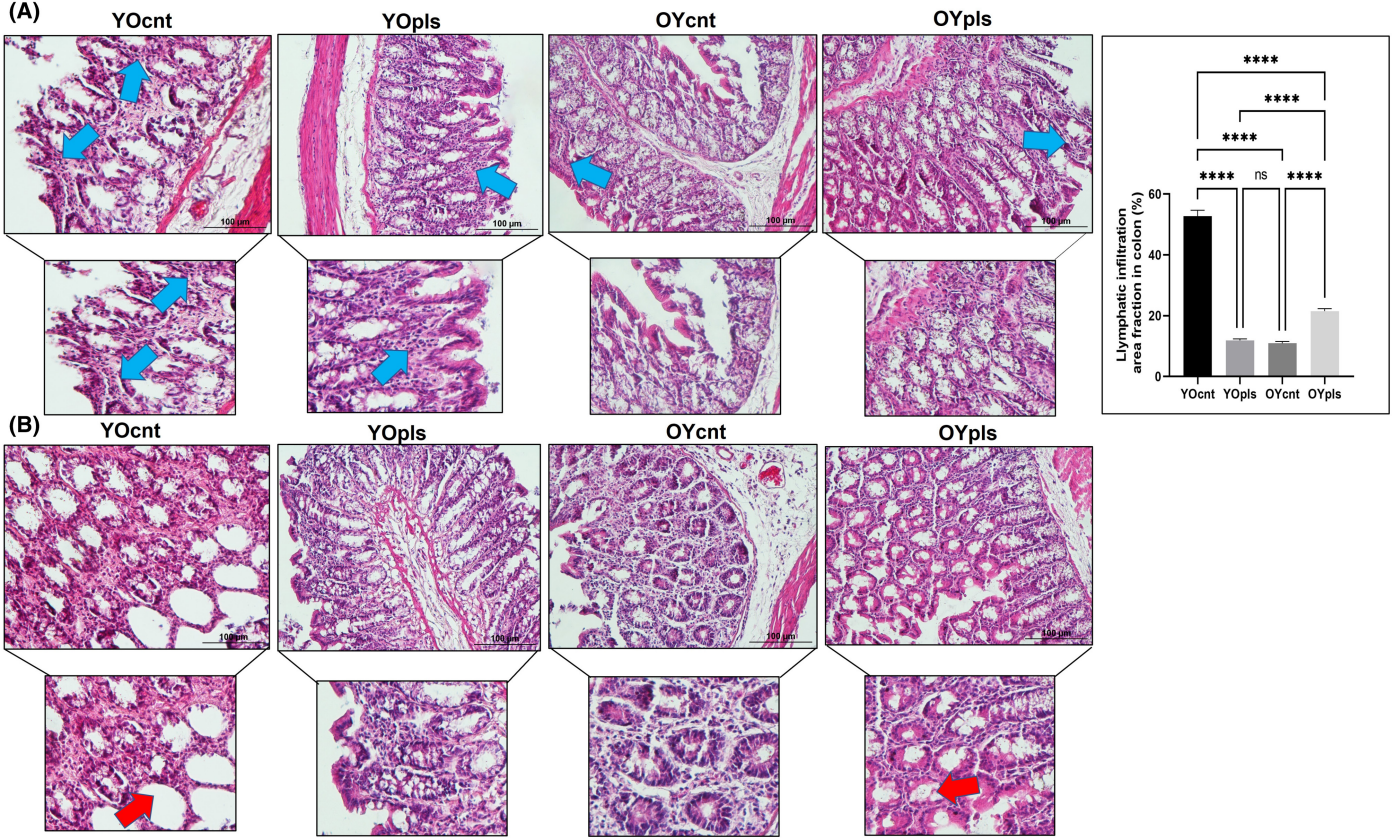
Representative images of haematoxylin and eosin stain staining and quantification of lymphatic infiltration area fraction (%) in all groups of colon sections. Aged rats’ colon show increased lymphatic infiltration, but young plasma received aged groups improved these histopathological alterations. Areas in the haematoxylin and eosin stain‐stained microphotographs of all groups were magnified in the photos to which they belonged to the area of interest. Values are expressed as mean ± SEM; *n* = 7 rats in each group. *p* ≤ 0.0001**** (nonparametric Mann–Whitney *U*‐test). Scale bar = 100 μm. YOcnt (aged control rats), OYcnt (young control rats), YOpls (young plasma recipient aged rats), and OYpls (aged plasma recipient young rats).

We explored the influence of old and young plasma treatments on ileum and colon tissues, assessing histopathological changes in serial sections. Young, healthy rats exhibited well‐preserved ileum and colon structures, including intact epithelium, muscle layers, and regular intestinal villi distribution (Figures 5A and 6A). Aged rats, in contrast, displayed prominent epithelial shedding in both the ileum and colon, leading to lamina propria exposure (Figures 5A and 6A). Significant reductions in the goblet and Paneth cell densities, vital for mucus production and gut health, were noted in the ileum of aged rats. This trend was reversed, however, when aged rats were treated with young plasma. Adverse effects, including goblet cell density decrease, mucosal necrosis, and glandular hyperplasia due to increased immune cell infiltration, were observed in the colon of aged rats, but these were mitigated with young plasma treatment (Figures 5B and 6B). Finally, we examined crypt cell proliferation in the small intestine, noting higher proliferation in the ileum of aged rats compared to the young. This increase was less pronounced in aged rats treated with young plasma (Figure 5C).

### 3.4 Young plasma ameliorates age‐related intestinal inflammation

Our study aimed to assess the inflammatory state in the ileum and colon tissues, as inflammation is a hallmark in aged rats. In aged rats, haematoxylin and eosin stain‐stained sections of these tissues revealed increased lymphatic infiltration, an inflammatory response marker. This infiltration was notably mitigated in aged rats that received young plasma (Figures 5A and 6A). We also observed decreased mucus secretion and abrasion of intestinal epithelium in aged rats compared to both young and young plasma‐treated aged rats. Young plasma‐treated aged rats showed mild inflammation and increased mucus secretion (Figures 5 and 6). This indicates a beneficial influence of young plasma on curtailing excessive inflammation and promoting mucus production in the ileum and colon. Conversely, aged plasma‐treated young rats displayed reduced mucus production, increasing lymphatic infiltration in the ileum and colon. Thus, our findings highlight young plasma's potential in countering the negative effects of ageing on intestinal health.

### 3.5 Quantitative histomorphometric analysis of ileum and colon

Ageing brings changes in the histological structure of ileum and colon tissues, impacting their height, length, thickness, and depth. The morphometric measurements for these tissues are detailed in Figures S5 and S6. Interestingly, young plasma transferred to aged rats and old plasma transferred to young rats showed significant length variations in the ileum and colon. We analysed villi height, crypt depth, and thickness of mucosa, submucosa, and total intestinal wall. Ageing increased villi height and the thickness of the submucosa, muscle layer, and total intestinal wall in aged rats (Figure S5A,D,E,F). Conversely, crypt depth and mucosa thickness reduced in aged rats receiving young plasma (Figure S5B,C). Thus, young plasma ameliorated all assessed histomorphometric parameters in aged rat ileum.

For colon tissues, we measured crypt depth and thicknesses of the mucosa, submucosa, muscle layer, and total wall (Figure S6). Aged rats showed reduced crypt depth compared to the young and young plasma‐received aged rats (Figure S6A). Young plasma also significantly decreased submucosa, muscle layer, and total wall thickness in aged rats (Figure S6C–E), positively affecting the colon's histological layers. However, aged plasma transferred to young rats increased submucosa and total wall thickness. The structural changes in young rats receiving aged plasma resembled aged colon histomorphological alterations.

### 3.6 Quantitative analysis of rat serosal mast cells density in the ileum and colon

We aimed to examine mast cells' contribution to age‐related intestinal injury. Violet‐purple mast cell densities were analysed in TB‐stained ileum and colon sections. In young rats, these cells were readily distinguishable, with homogeneous granules. However, aged rats had a predominantly higher density of mast cells in the serosa layers of both the ileum and colon. This density was less in young plasma‐received aged rats and young groups (Figures S7 and S8). The increased mast cell density in aged rats also showed a higher presence of granules, establishing a correlation with intestinal inflammation. However, aged rats receiving young plasma displayed a significant reduction in mast cell density, emphasizing the potential of young plasma to mitigate mast cell proliferation and intestinal inflammation.

### 3.7 Immunochemical analysis of inhibitory effects of young plasma on inflammatory mediators inducible TNF‐α and COX‐2 in aged ileum and colon

**FIGURE 7:**
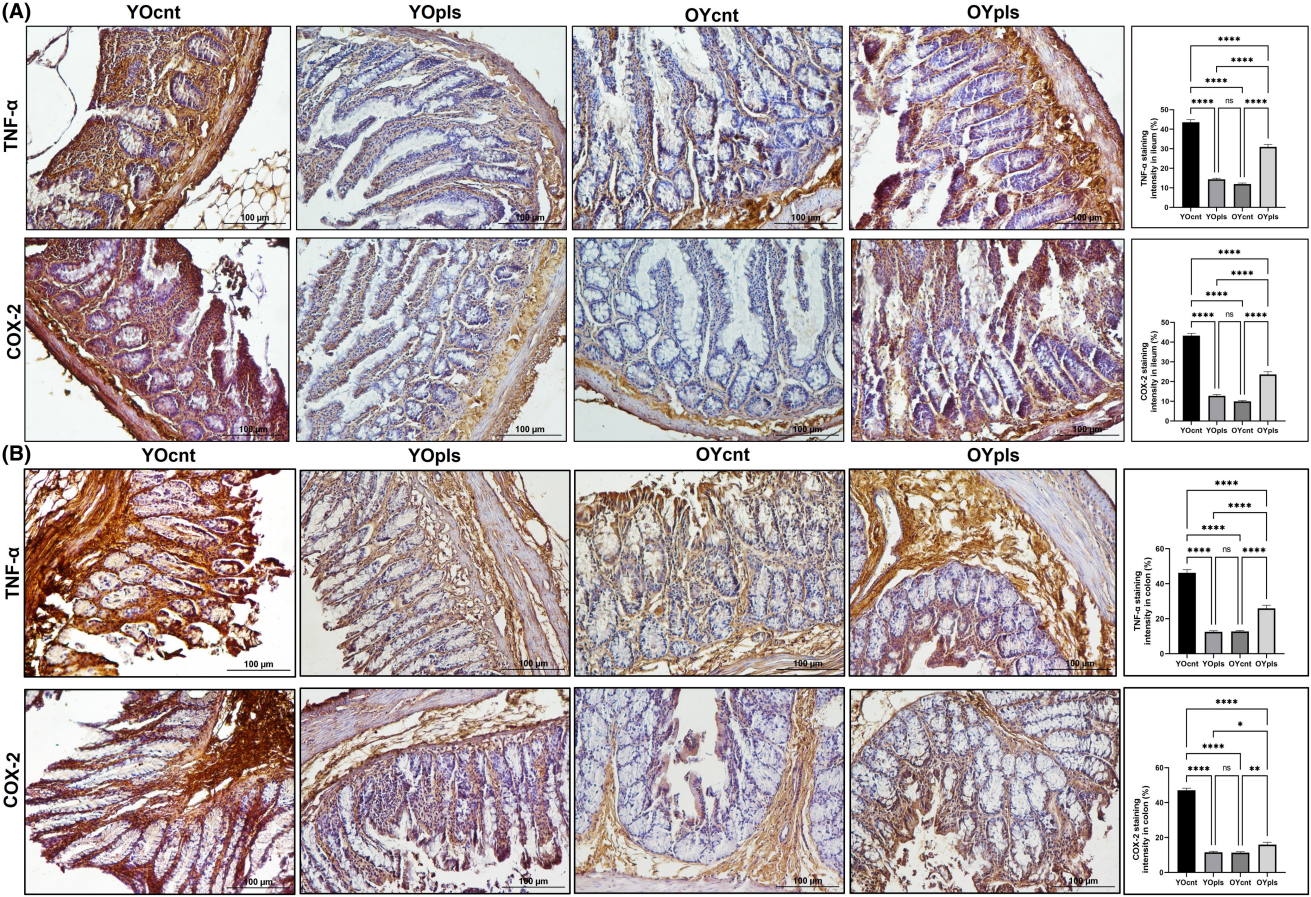
TNF‐α and COX‐2 staining intensity (A) ileum, and (B) colon. IHC staining images of TNF‐α and COX‐2 expression in the rat ileum and colon tissues. Graphs of TNF‐α and COX‐2 staining intensity in the ileum colon as measured in ImageJ (FIJI). YOcnt (aged control rats), OYcnt (young control rats), YOpls (young plasma recipient aged rats), and OYpls (aged plasma recipient young rats); (****p ≤ 0.0001) (IHC staining, Scale bar: 100 μm). COX‐2, cyclooxygenase‐2; IHC, immunohistochemical; TNF‐α, tumour necrosis factor‐alpha.

Our study also examined the effect of old and young plasma on the expression of inflammatory enzymes TNF‐α and COX‐2 in the ileum and colon tissues of aged and young rats (Figures 7A and 7B). These enzymes were less expressed in aged rats receiving young plasma compared to other groups. TNF‐α, primarily located in the mucosa and lamina propria, was significantly increased in the aged rats but reduced in response to young plasma. This reduction was significant in an age‐dependent manner (*p* ≤ 0.0001 ****). Young plasma also significantly decreased the expression of COX‐2 in the ileum and colon tissues of aged rats. In contrast, the expression of COX‐2 increased in the aged plasma‐received young rats and was scarcely found in the young control groups. Its expression was elevated in the surface epithelium cells and inflammatory infiltrate cells in the aged rats' group.

## 4 DISCUSSION

Ageing, characterized by gradual physiological and molecular changes, significantly impacts the function of vital barriers such as the intestinal epithelium.^27^, ^28^, ^29^ Our study revealed these effects in aged rat ileum and colon tissues, noting increased epithelial shedding and inflammation, reduced cell density, and morphometric alterations. Interestingly, we found young plasma could mitigate these age‐related changes, demonstrating therapeutic potential. Similar rejuvenating effects were seen in liver and testicular tissues.^9^, ^30^ Conversely, aged plasma negatively affected young rats.

The ageing process exacerbates inflammation and protein alterations, which incite the generation of reactive oxygen species. This phenomenon further propels protein modifications and cultivates environments conducive to lymphatic infiltration, a correlation that our results affirm.^31^, ^32^ Our study showed that the administration of young plasma to aged rats resulted in a decline in lymphatic infiltration density,^28^, ^33^ shedding light on its anti‐inflammatory potential. We further investigated changes in mast cell densities in the ileum and colon and the expression levels of inflammatory mediators such as TNF‐α and COX‐2. Our findings highlighted that aged rats experienced an increase in serosal mast cell densities and upregulated expression of TNF‐α and COX‐2, leading to heightened oxidative stress and inflammation. Remarkably, the introduction of young plasma mitigated these adverse effects, indicating its therapeutic potential for combating age‐induced inflammation. Furthermore, we note that young blood plasma, enriched with anti‐ageing metabolites such as GDF11, TIMP2, Oxytocin, and Osteocalcin, exhibits rejuvenating properties in aged tissues.^34^ This claim is substantiated by observed reductions in various ageing biomarkers, including ferric‐reducing ability, plasma membrane redox system activity, glutathione, malondialdehyde levels, and protein carbonylation (PCO), following young plasma administration.^1^ Particularly, the significant decrease in the spectrochemical band PCO, a well‐established indicator of oxidative stress‐related disorders,^35^ in the ileum and colon tissues of aged rats post‐young plasma treatment attests to this rejuvenating effect. In contrast, young rats subjected to aged plasma demonstrate the deleterious impact of ageing on plasma. These findings illuminate the rejuvenating capacity of young plasma and its potential role in future anti‐ageing interventions.

Our machine learning analysis correlates with these findings, revealing a younger lipid biomolecule profile in both ileum and colon tissues post‐young plasma transfer. Furthermore, an increased presence of mitotic figures in aged tissue samples indicates a proliferation within the stem cell pool, suggesting that ageing‐induced cellular damage triggers stem cell activation. However, spectrochemical analyses reveal differential responses to plasma transfer in the ileum and colon tissues, given their distinct roles in digestion.^36^ Unsaturated fatty acids have been shown to enhance endoplasmic reticulum function, while saturated fatty acids impair it by reducing membrane flexibility.^37^ Therefore, the decrease in saturated lipid content in the ileum tissue following young plasma transfer could be indicative of cellular regeneration. Conversely, the adverse effect of old plasma transfer appears to be an increase in the saturated lipid content in the young colon tissue. These findings suggest that young plasma transfer has restorative effects on the lipid composition of the ileum and colon tissues, with more significant changes observed in the ileum. However, protein phosphorylation in the ileum and colon decreased following young plasma transfer and increased after old plasma administration. Protein phosphorylation is a crucial post‐translational modification that influences a wide range of cellular processes, including gene expression, metabolism, differentiation, and cell cycle regulation.^38^ As protein function can be differentially impacted by modifications at distinct sites,^39^ comprehensive proteomic analysis is needed to fully interpret these phosphorylation changes.

Ageing undermines intestinal epithelial regeneration and barrier integrity, as demonstrated in both animals and humans.^40^, ^41^ Concurrently, the gut microbiota deteriorates with ageing, leading to dysbiosis, a condition linked to diseases such as irritable bowel syndrome and leaky gut syndrome.^42^, ^43^, ^44^ The gut microbiota and the intestinal epithelium are separated merely by a mucus layer.^45^ This mucosal barrier comprises mucin, antimicrobial peptides, and IgA, secreted by goblet cells, Paneth cells, and plasma cells, respectively. They form a shield against microbial adherence to the epithelium.^46^ Antimicrobial peptides, particularly those from Paneth cells, destroy pathogens and control gut microbiota diversity, thereby preserving the integrity of the intestinal tissue.^47^, ^48^ Our study reinforces these observations while unveiling new insights into the effects of young plasma transfer. Specifically, we found an increase in the number of goblets and Paneth cells in aged rats after young plasma transfer. These changes are in alignment with the alterations we observed in the gut microbiota of the rats used in this study.^49^ We noted a significant increase in microbiota diversity (alpha diversity indices) and a normalization of the Firmicutes to Bacteroidetes ratio, an indicator of healthy gut microbiota, in aged rats after young plasma transfer. These bacterial groups are known to produce short‐chain fatty acids, essential nutrients for intestinal epithelial cells.^50^ We also confirmed this in a recent study where young plasma administration enhanced gut microbiota diversity in 12‐month‐old Wistar rats.^15^ Therefore, our data collectively highlight the potential rejuvenating effects of young plasma on gut health in aged subjects.

In conclusion, this study shows that young plasma has rejuvenating effects on aged ileum and colon tissues, suggesting its therapeutic potential for intestinal ageing. Machine learning analysis reveals similarbiomolecular profiles between aged tissues treated with young plasma and younger tissues. IR spectroscopy analyses detected changes in various spectrochemical bands, indicating alterations in lipid, protein, and nucleic acid profiles in both tissues. The efficiency of young plasma in restoring biomolecule profiles was more pronounced in the ileum compared to the colon. Both tissues exhibited significant changes, but the ileum demonstrated a more distinct response to plasma exchange. Quantitative changes in spectrochemical indices revealed alterations in saturated lipid, lipid/protein, nucleic acid/protein, protein carbonylation, protein phosphorylation, and glucose/protein content in both tissues. However, the colon tissue displayed more significant changes in these indices after aged plasma transfer compared to the ileum. Histopathological and immunohistochemical assessments demonstrate that young plasma can restore tissue architecture and function, including goblet and Paneth cell densities, reduce inflammation and decrease mast cell density. The expression levels of inflammatory mediators TNF‐α and COX‐2 significantly decrease in aged rats treated with young plasma. Conversely, young rat tissues treated with aged plasma show accelerated ageing. These findings highlight the therapeutic potential of young plasma in alleviating age‐related intestinal alterations and inflammation. Further research is needed, particularly in different intestinal regions' response to plasma exchange, to gain insights into gut health and ageing. This study contributes to our understanding of the interplay between ageing, gut health, and plasma composition, informing the development of novel therapeutic strategies for improving overall health and lifespan.

## AUTHOR CONTRIBUTIONS

**Taha Ceylani:** Conceptualization (equal); data curation (equal); formal analysis (equal); funding acquisition (equal); investigation (equal); methodology (equal); resources (equal); software (equal); supervision (equal); validation (equal); visualization (equal); writing – original draft (equal); writing – review and editing (equal). **Hikmet Taner Teker:** Conceptualization (equal); data curation (equal); formal analysis (equal); funding acquisition (equal); investigation (equal); methodology (equal); resources (equal); software (equal); supervision (equal); validation (equal); visualization (equal); writing – original draft (equal); writing – review and editing (equal). **Seda Keskin:** Investigation (equal); methodology (equal); resources (equal); software (equal); supervision (equal); validation (equal); visualization (equal); writing – original draft (equal). **Gizem Samgane:** Methodology (equal); software (equal); supervision (equal); validation (equal); visualization (equal). **Eda Acikgoz:** Methodology (equal); resources (equal); software (equal); supervision (equal); validation (equal); visualization (equal); writing – original draft (equal). **Rafig Gurbanov:** Data curation (equal); formal analysis (equal); funding acquisition (equal); investigation (equal); methodology (equal); resources (equal); software (equal); supervision (equal); validation (equal); visualization (equal); writing – original draft (equal); writing – review and editing (equal).

## FUNDING INFORMATION

This research received no specific grant from any funding agency in the public, commercial, or not‐for‐profit sectors.

## CONFLICT OF INTEREST STATEMENT

The authors have declared that no competing interests exist.

## CONSENT

Not applicable.

## CONSENT FOR PUBLICATION

Not applicable.

## Supporting information


Figure S1.
Figure S2.
Figure S3.
Figure S4.
Figure S5.
Figure S6.
Figure S7.
Figure S8.

Table S1.
Table S2.
Table S3.
Table S4.
Table S5.
Table S6.
Table S7.
Table S8.
Table S9.
Table S10.
Table S11.
Table S12.
Table S13.
Table S14.
Table S15.
Table S16.
Table S17.
Table S18.

## Data Availability

The datasets generated during and/or analysed during the current study are available from the corresponding author upon reasonable request.
