# Supplementary material for: The rejuvenating influence of young plasma on aged intestine
Source: J Cell Mol Med. 2023 Aug 23;27(18):2804–16. doi: 10.1111/jcmm.17926 (PMC10494294; doi:10.1111/jcmm.17926)
Supplement: Supplementary file 1 — Figure S1. Figure S2. Figure S3. Figure S4. Figure S5. Figure S6. Figure S7. Figure S8. [file JCMM-27-2804-s002.docx]

**Supplementary Figures**


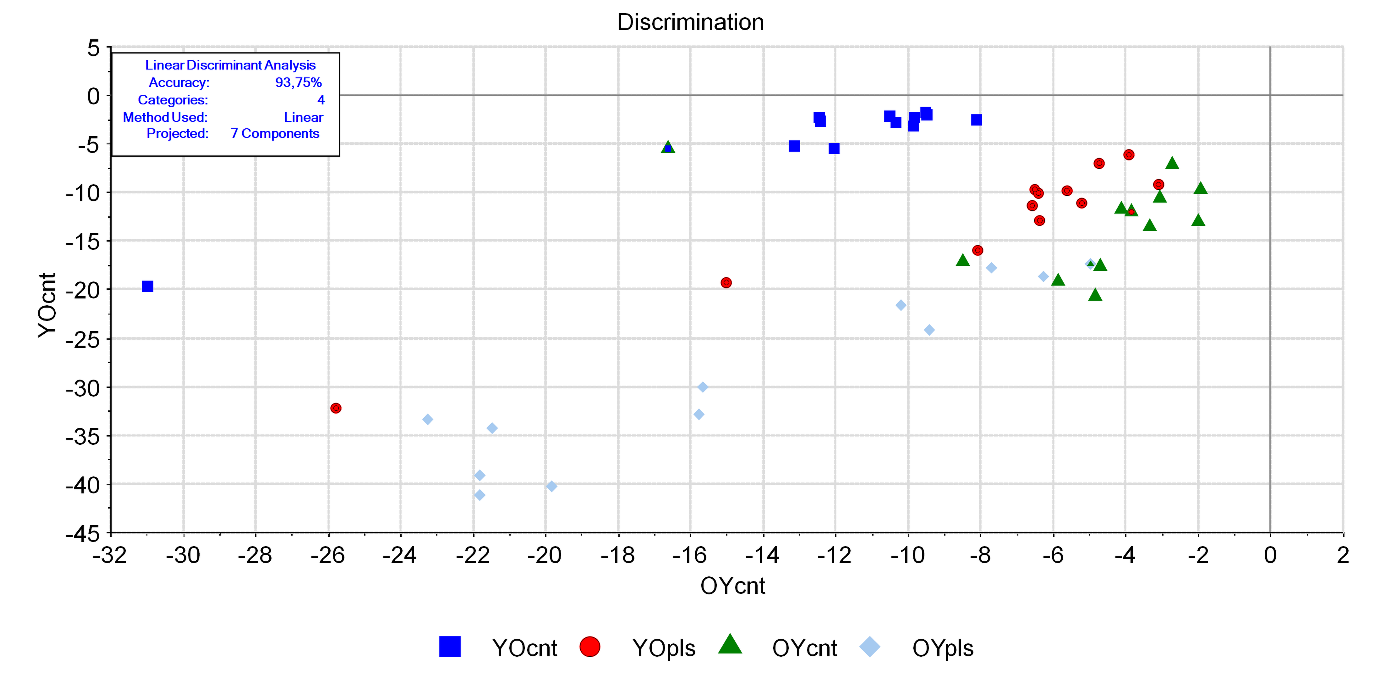


**Fig. S1** LDA discrimination plot for ileum samples in the full spectral region (4000-650 cm^-1^). YOcnt (aged control rats), OYcnt (young control rats), YOpls (young plasma recipient aged rats), OYpls (aged plasma recipient young rats)


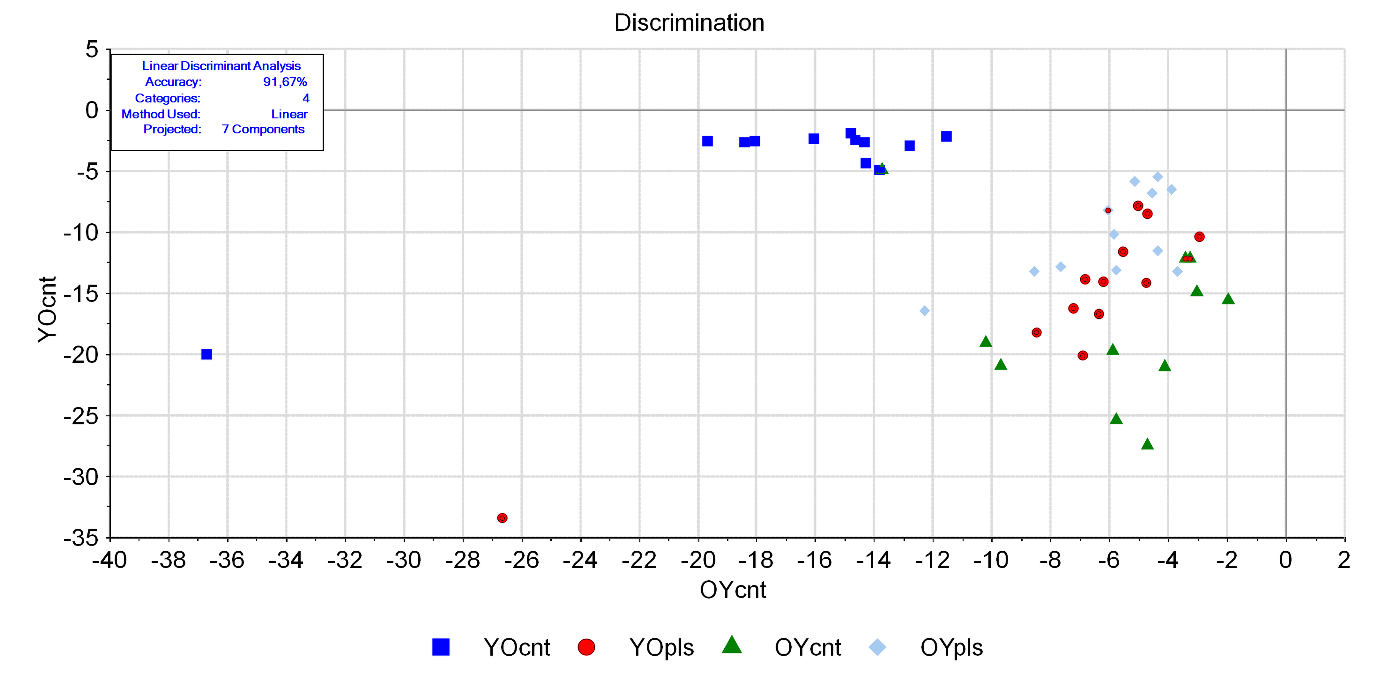


**Fig. S2** LDA discrimination plot for ileum samples in the spectral region of the nucleic acids and polysaccharides (1200-650 cm^−1^). YOcnt (aged control rats), OYcnt (young control rats), YOpls (young plasma recipient aged rats), OYpls (aged plasma recipient young rats)


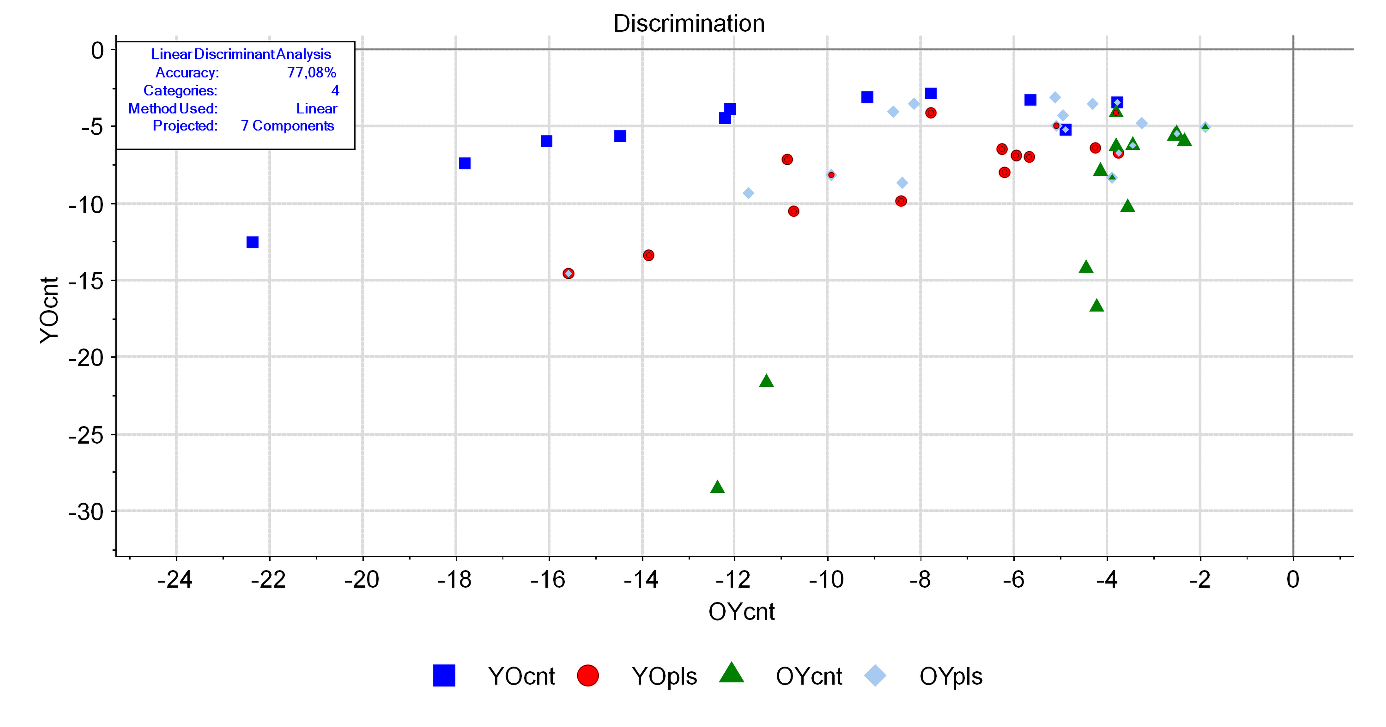


**Fig. S3** LDA discrimination plot for colon samples in the full (4000-650 cm^-1^) spectral region. YOcnt (aged control rats), OYcnt (young control rats), YOpls (young plasma recipient aged rats), OYpls (aged plasma recipient young rats)


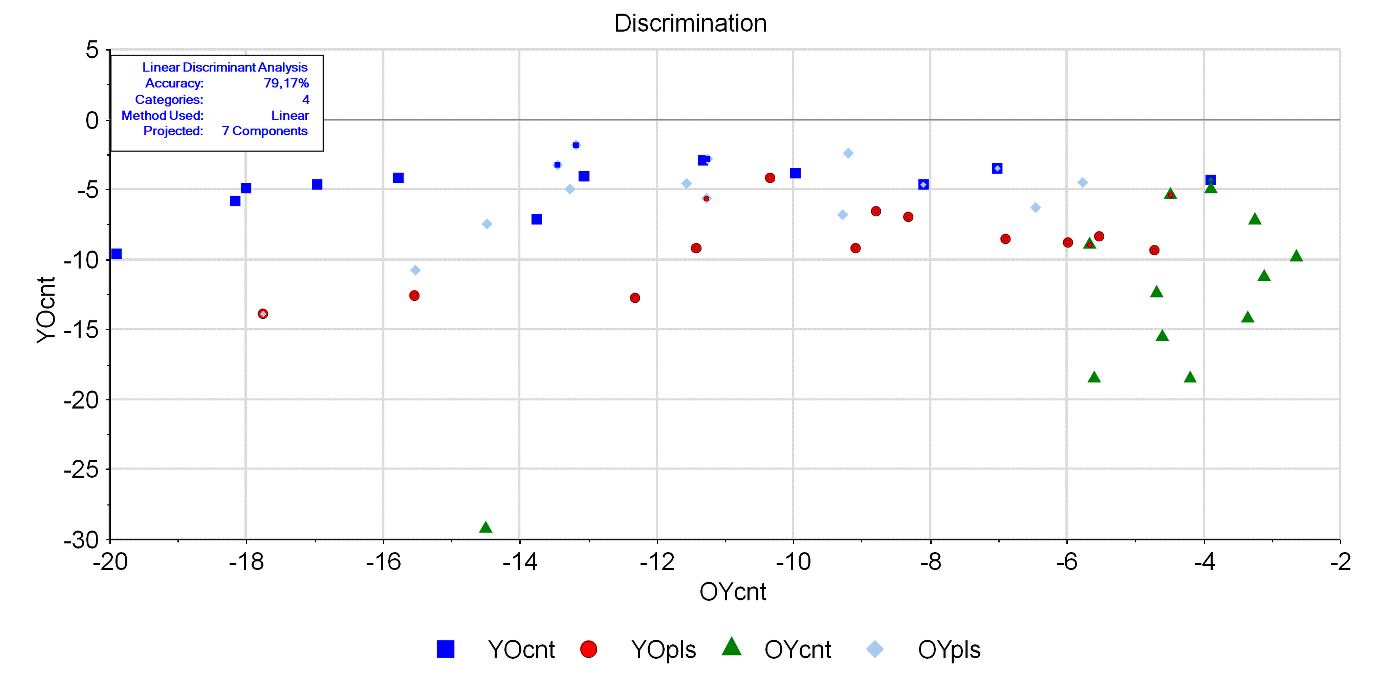


**Fig. S4** LDA discrimination plot for colon samples in the spectral region of the nucleic acids and polysaccharides (1200-650 cm^−1^). YOcnt (aged control rats), OYcnt (young control rats), YOpls (young plasma recipient aged rats), OYpls (aged plasma recipient young rats)

**
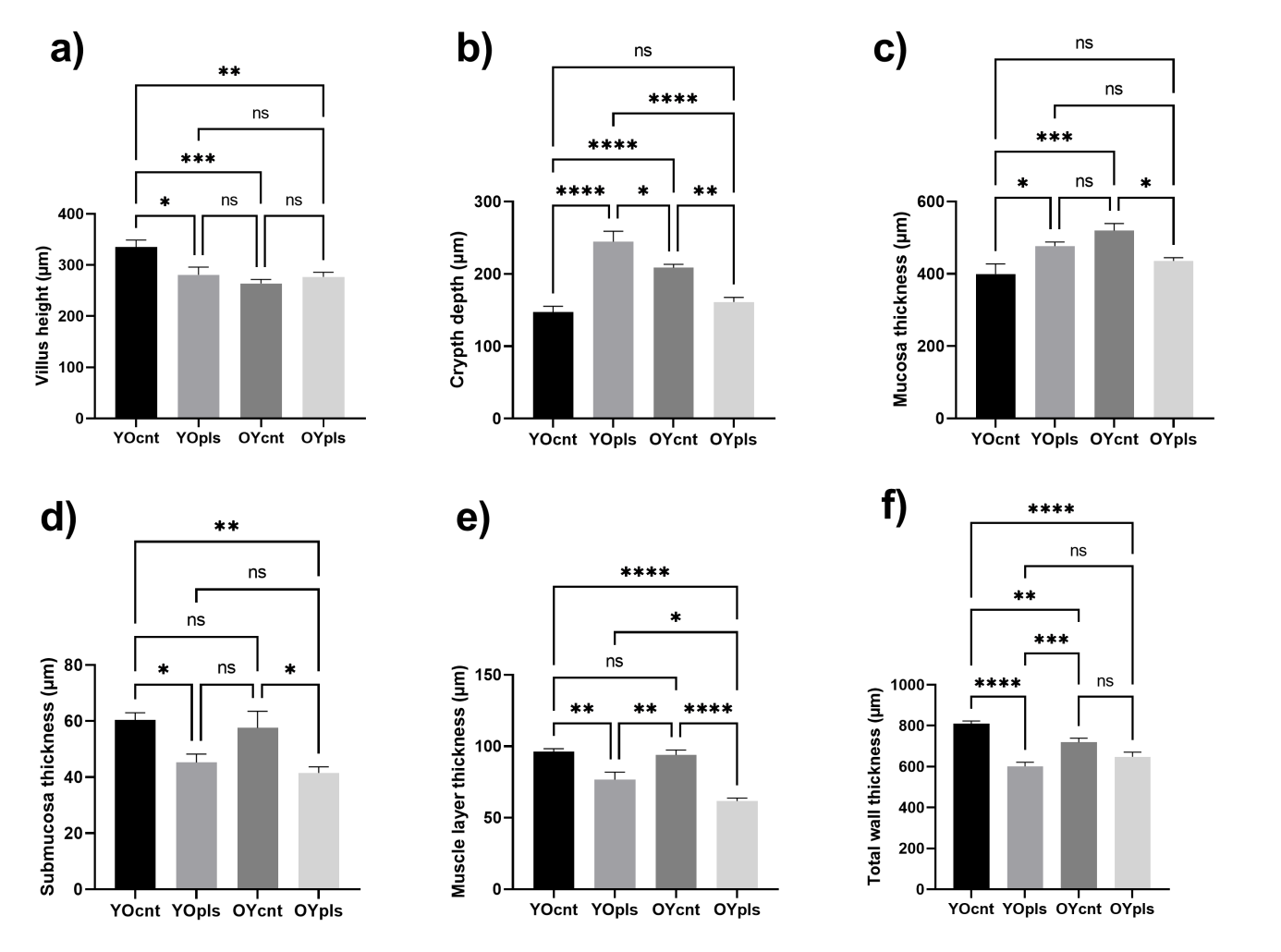
**

**Fig S5.** Histomorphometric analysis of intestinal parameters of rat ileum tissues evaluated in all groups. Measurements are shown as (a) villus height (µm), (b) crypth depth (µm), (c) mucosa thickness (µm), (d) submucosa thickness (µm), (e) mucosa layer thickness (µm), and (f) intestinal total wall thickness (µm). YOcnt (aged control rats), OYcnt (young control rats), YOpls (young plasma recipient aged rats), OYpls (aged plasma recipient young rats). p ≤ 0.05 *, p ≤ 0.01 **, p ≤ 0.001 ***, and p ≤ 0.0001 **** (one-way ANOVA test with Tukey's post-hoc test). Results are presented as mean ± SEM (standard error of the mean).

**
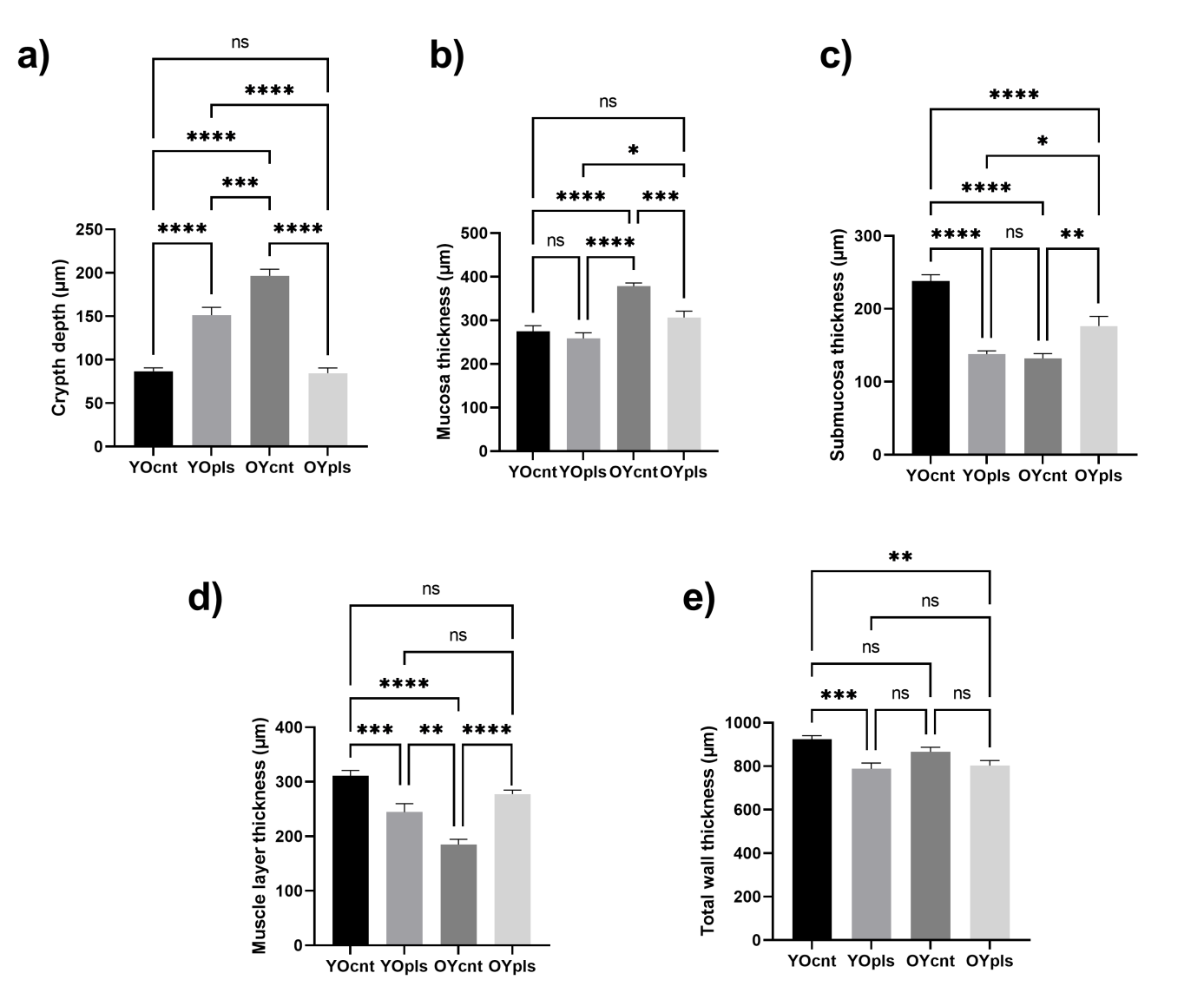
**

**Fig S6.** Histomorphometric analysis of intestinal parameters of rat colon tissues evaluated in all groups. Measurements are shown as (a) crypth depth (µm), (b) mucosa thickness(µm), (c) submucosa thickness (µm), (d) mucosa layer thickness (µm), and (e) intestinal total wall thickness (µm). YOcnt (aged control rats), OYcnt (young control rats), YOpls (young plasma recipient aged rats), OYpls (aged plasma recipient young rats). p ≤ 0.05 *, p ≤ 0.01 **, p ≤ 0.001 ***, and p ≤ 0.0001 **** (one-way ANOVA test with Tukey's post-hoc test). Results are presented as mean ± SEM (standard error of the mean).


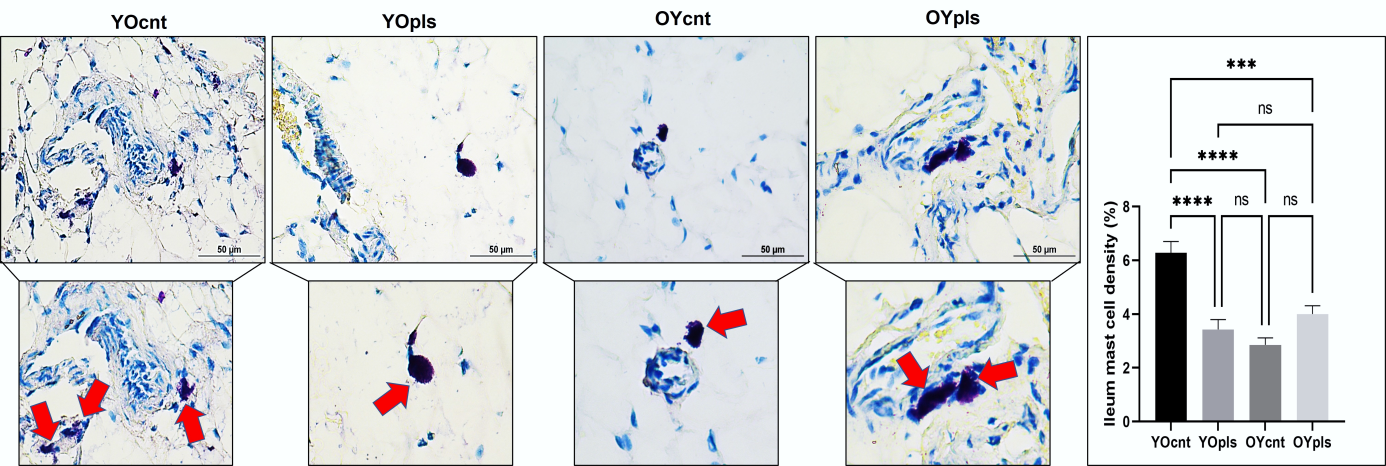


**Fig.S7.** Representative toluidine blue staining images of ileum tissue with quantification of mast cell density area fraction (%) in each group. Red arrows show intestinal serosal mast cells. Graph of TB staining serosal mast cells intensity in the rat ileum as measured in ImageJ (FIJI). Values are expressed as mean ±SEM; n = 7 rats in each group. p ≤ 0.001 *** and p ≤ 0.0001 **** (nonparametric Mann‐Whitney U test). Scale bar = 50 µm. YOcnt (aged control rats), OYcnt (young control rats), YOpls (young plasma recipient aged rats), OYpls (aged plasma recipient young rats).


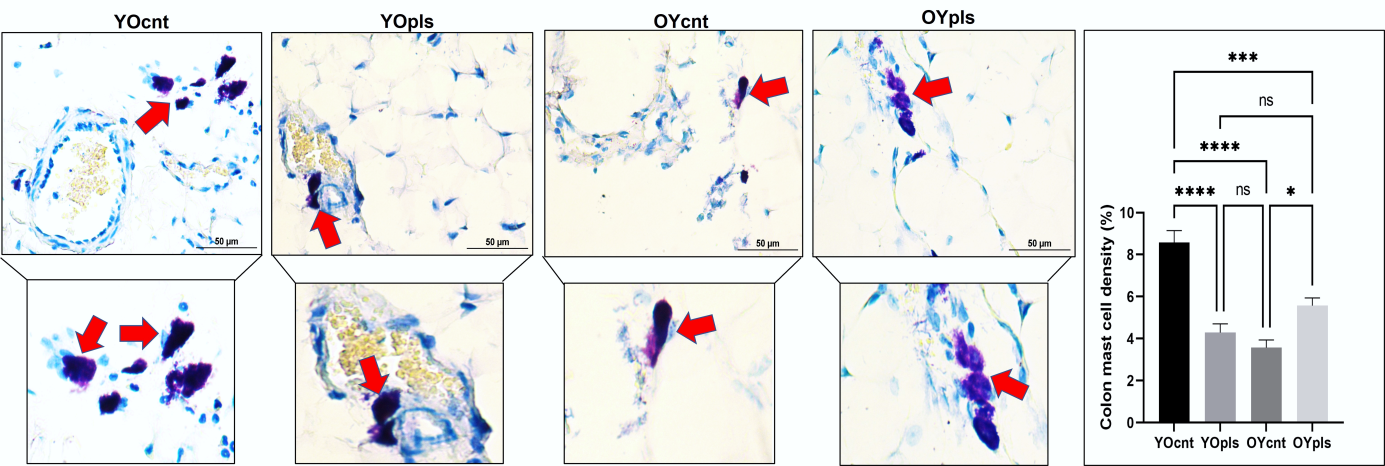


**Fig.S8.** Representative toluidine blue staining images of colon tissue with quantification of mast cell density area fraction (%) in each group. Red arrows show intestinal serosal mast cells. Graph of TB staining serosal mast cells intensity in the rat colon as measured in ImageJ (FIJI). Values are expressed as mean ±SEM; n = 7 rats in each group. p ≤ 0.05 *, p ≤ 0.001 *** and p ≤ 0.0001 **** (nonparametric Mann‐Whitney U test) vs. control. Scale bar = 50 µm. YOcnt (aged control rats), OYcnt (young control rats), YOpls (young plasma recipient aged rats), OYpls (aged plasma recipient young rats).
