# Supplementary material for: The rejuvenating influence of young plasma on aged intestine
Source: J Cell Mol Med. 2023 Aug 23;27(18):2804–16. doi: 10.1111/jcmm.17926 (PMC10494294; doi:10.1111/jcmm.17926)
Supplement: Supplementary file 2 — Table S1. Table S2. Table S3. Table S4. Table S5. Table S6. Table S7. Table S8. Table S9. Table S10. Table S11. Table S12. Table S13. Table S14. Table S15. Table S16. Table S17. Table S18. [file JCMM-27-2804-s001.docx]

**Supplementary Tables**

**Table S1** LDA confusion matrix for ileum samples in lipid (3000-2700 cm^-1^) spectral region. YOcnt (aged control rats), OYcnt (young control rats), YOpls (young plasma recipient aged rats), OYpls (aged plasma recipient young rats)

| **Confusion matrix** | **Actual** | **YOCNT** | **YOPLS** | **OYCNT** | **OYPLS** |
| --- | --- | --- | --- | --- | --- |
| **Predicted** |  | **1** | **2** | **3** | **4** |
| **YOCNT** | **1** | **12** | 0 | 1 | 0 |
| **YOPLS** | **2** | 0 | **10** | 1 | 0 |
| **OYCNT** | **3** | 0 | 2 | **10** | 0 |
| **OYPLS** | **4** | 0 | 0 | 0 | **12** |

**Table S2** LDA prediction matrix for ileum samples in lipid (3000-2700 cm^-1^) spectral region. YOcnt (aged control rats), OYcnt (young control rats), YOpls (young plasma recipient aged rats), OYpls (aged plasma recipient young rats)

| **Prediction Matrix** | **YOCNT** | **YOPLS** | **OYCNT** | **OYPLS** | **Predicted** |
| --- | --- | --- | --- | --- | --- |
| Sample | 1 | 2 | 3 | 4 | 5 |
| 1 | -2,4862 | -15,5916 | -9,6872 | -10,0618 | YOCNT |
| 2 | -3,8163 | -11,8961 | -6,8285 | -10,0444 | YOCNT |
| 3 | -3,1777 | -24,1758 | -16,2745 | -9,7476 | YOCNT |
| 4 | -2,6805 | -16,7805 | -10,6945 | -6,5213 | YOCNT |
| 5 | -2,4097 | -16,7813 | -11,0544 | -13,5816 | YOCNT |
| 6 | -2,2492 | -17,4024 | -10,7543 | -9,0620 | YOCNT |
| 7 | -19,7544 | -38,9271 | -33,4088 | -31,8565 | YOCNT |
| 8 | -3,1571 | -15,0599 | -11,6411 | -15,1658 | YOCNT |
| 9 | -6,8692 | -17,9034 | -11,3331 | -18,5910 | YOCNT |
| 10 | -1,8393 | -14,2280 | -8,9024 | -10,3789 | YOCNT |
| 11 | -2,2722 | -16,4321 | -10,8588 | -8,9363 | YOCNT |
| 12 | -2,1364 | -12,0753 | -7,6528 | -10,3422 | YOCNT |
| 13 | -36,3386 | -17,5668 | -20,3697 | -56,5583 | YOPLS |
| 14 | -16,4870 | -4,8204 | -4,3591 | -24,9042 | OYCNT |
| 15 | -5,3999 | -5,6793 | -3,3980 | -17,7262 | OYCNT |
| 16 | -11,9558 | -2,0551 | -2,7952 | -24,9703 | YOPLS |
| 17 | -23,3930 | -2,9962 | -9,2480 | -45,4462 | YOPLS |
| 18 | -25,9160 | -3,2606 | -9,4561 | -46,6714 | YOPLS |
| 19 | -20,7298 | -2,1628 | -6,2275 | -38,2928 | YOPLS |
| 20 | -20,0519 | -2,2267 | -6,8925 | -39,1931 | YOPLS |
| 21 | -16,8983 | -2,5889 | -5,9557 | -37,8773 | YOPLS |
| 22 | -21,2628 | -2,6524 | -5,4860 | -38,0144 | YOPLS |
| 23 | -11,1269 | -2,0741 | -3,0178 | -25,8334 | YOPLS |
| 24 | -7,1457 | -4,2172 | -4,7049 | -22,0634 | YOPLS |
| 25 | -11,7541 | -7,5627 | -2,8892 | -18,5314 | OYCNT |
| 26 | -5,7513 | -5,3876 | -2,4860 | -14,3278 | OYCNT |
| 27 | -9,5754 | -6,2560 | -2,0487 | -19,0532 | OYCNT |
| 28 | -15,8319 | -14,5397 | -9,0050 | -25,8242 | OYCNT |
| 29 | -10,1577 | -3,5807 | -3,0476 | -25,5747 | OYCNT |
| 30 | -16,1584 | -10,2423 | -5,2157 | -20,0081 | OYCNT |
| 31 | -8,6696 | -3,5707 | -2,4240 | -18,5423 | OYCNT |
| 32 | -14,8198 | -6,7443 | -5,3946 | -27,9762 | OYCNT |
| 33 | -14,8417 | -2,7123 | -5,0270 | -29,4468 | YOPLS |
| 34 | -24,9262 | -6,9963 | -6,0943 | -35,5972 | OYCNT |
| 35 | -12,2465 | -6,3571 | -4,4530 | -26,3814 | OYCNT |
| 36 | -4,8092 | -8,9603 | -5,2147 | -10,0767 | YOCNT |
| 37 | -9,9543 | -37,4081 | -24,1346 | -9,8950 | OYPLS |
| 38 | -7,7957 | -28,3979 | -16,3725 | -2,8853 | OYPLS |
| 39 | -4,0142 | -21,1833 | -13,0053 | -3,9721 | OYPLS |
| 40 | -6,3844 | -26,2828 | -16,5197 | -2,3958 | OYPLS |
| 41 | -12,9517 | -31,3326 | -18,2662 | -2,4777 | OYPLS |
| 42 | -31,8738 | -53,5719 | -39,5479 | -19,0246 | OYPLS |
| 43 | -17,5388 | -46,4541 | -29,9718 | -3,9290 | OYPLS |
| 44 | -12,7197 | -33,9973 | -22,0082 | -2,0668 | OYPLS |
| 45 | -18,6324 | -43,4014 | -28,3352 | -3,2227 | OYPLS |
| 46 | -18,9924 | -37,3181 | -23,6173 | -4,3059 | OYPLS |
| 47 | -12,1495 | -34,3819 | -24,0721 | -3,4271 | OYPLS |
| 48 | -10,5274 | -33,6147 | -24,2830 | -4,4917 | OYPLS |

**Table S3** LDA confusion matrix for ileum samples in protein (1700-1500 cm^-1^) spectral region. YOcnt (aged control rats), OYcnt (young control rats), YOpls (young plasma recipient aged rats), OYpls (aged plasma recipient young rats)

| **Confusion matrix** | **Actual** | **YOCNT** | **YOPLS** | **OYCNT** | **OYPLS** |
| --- | --- | --- | --- | --- | --- |
| **Predicted** |  | **1** | **2** | **3** | **4** |
| **YOCNT** | **1** | **9** | 0 | 0 | 3 |
| **YOPLS** | **2** | 0 | **12** | 1 | 0 |
| **OYCNT** | **3** | 0 | 0 | **11** | 0 |
| **OYPLS** | **4** | 3 | 0 | 0 | **9** |

**Table S4** LDA prediction matrix for ileum samples in protein (1700-1500 cm^-1^) spectral region. YOcnt (aged control rats), OYcnt (young control rats), YOpls (young plasma recipient aged rats), OYpls (aged plasma recipient young rats)

| **Prediction Matrix** | **YOCNT** | **YOPLS** | **OYCNT** | **OYPLS** | **Predicted** |
| --- | --- | --- | --- | --- | --- |
| Sample | 1 | 2 | 3 | 4 | 5 |
| 1 | -2,918 | -6,2433 | -7,5256 | -3,2028 | YOCNT |
| 2 | -5,5309 | -6,2879 | -10,968 | -5,3482 | OYPLS |
| 3 | -2,4638 | -10,258 | -11,243 | -3,7028 | YOCNT |
| 4 | -2,1065 | -10,177 | -13,388 | -3,2065 | YOCNT |
| 5 | -3,0963 | -14,599 | -18,628 | -3,0319 | OYPLS |
| 6 | -2,8759 | -9,7681 | -9,1932 | -2,7295 | OYPLS |
| 7 | -8,0536 | -11,154 | -17,926 | -8,8068 | YOCNT |
| 8 | -4,3318 | -11,457 | -17,093 | -6,4595 | YOCNT |
| 9 | -3,5126 | -8,5202 | -7,8394 | -4,4747 | YOCNT |
| 10 | -2,3934 | -14,709 | -17,235 | -2,8347 | YOCNT |
| 11 | -3,3895 | -16,821 | -18,137 | -3,6776 | YOCNT |
| 12 | -1,715 | -8,9134 | -12,646 | -2,8228 | YOCNT |
| 13 | -29,203 | -18,96 | -21,297 | -27,745 | YOPLS |
| 14 | -19,826 | -7,5643 | -11,743 | -20,638 | YOPLS |
| 15 | -9,3442 | -2,6855 | -4,2686 | -11,856 | YOPLS |
| 16 | -8,2662 | -1,6846 | -3,2835 | -9,6658 | YOPLS |
| 17 | -4,9938 | -2,8874 | -5,3097 | -6,7191 | YOPLS |
| 18 | -8,7744 | -1,894 | -6,2242 | -9,9298 | YOPLS |
| 19 | -12,774 | -2,3871 | -5,9997 | -14,824 | YOPLS |
| 20 | -5,674 | -2,6784 | -8,1513 | -7,106 | YOPLS |
| 21 | -9,906 | -2,5647 | -4,8848 | -12,136 | YOPLS |
| 22 | -22,581 | -11,693 | -17,608 | -23,461 | YOPLS |
| 23 | -9,1531 | -2,1287 | -4,1689 | -10,805 | YOPLS |
| 24 | -6,1114 | -2,958 | -5,8733 | -8,6507 | YOPLS |
| 25 | -19,657 | -7,8273 | -3,0725 | -19,885 | OYCNT |
| 26 | -15,015 | -4,3043 | -2,5308 | -16,468 | OYCNT |
| 27 | -16,459 | -7,6265 | -2,953 | -16,968 | OYCNT |
| 28 | -27,855 | -17,806 | -9,5605 | -29,372 | OYCNT |
| 29 | -14,303 | -9,6311 | -5,2241 | -15,034 | OYCNT |
| 30 | -19,373 | -13,715 | -6,3344 | -16,369 | OYCNT |
| 31 | -5,4694 | -2,161 | -5,2969 | -6,279 | YOPLS |
| 32 | -12,867 | -3,5354 | -2,8786 | -14,72 | OYCNT |
| 33 | -6,7467 | -4,2854 | -3,0135 | -6,8267 | OYCNT |
| 34 | -15,05 | -6,6314 | -3,3111 | -13,873 | OYCNT |
| 35 | -14,806 | -9,1306 | -4,3266 | -15,313 | OYCNT |
| 36 | -7,0622 | -7,3005 | -6,7267 | -7,0872 | OYCNT |
| 37 | -8,3879 | -16,065 | -13,768 | -7,9021 | OYPLS |
| 38 | -3,6966 | -5,188 | -7,9499 | -3,3151 | OYPLS |
| 39 | -2,7936 | -7,4512 | -10,036 | -3,6327 | YOCNT |
| 40 | -2,7232 | -9,2287 | -13,165 | -2,6438 | OYPLS |
| 41 | -4,8709 | -12,23 | -10,699 | -3,2505 | OYPLS |
| 42 | -18,791 | -34,738 | -37,761 | -18,137 | OYPLS |
| 43 | -7,0309 | -19,906 | -17,7 | -4,2802 | OYPLS |
| 44 | -3,0691 | -10,422 | -13,469 | -2,1991 | OYPLS |
| 45 | -8,0095 | -22,21 | -22,575 | -5,3908 | OYPLS |
| 46 | -4,1653 | -10,275 | -10,229 | -2,3943 | OYPLS |
| 47 | -3,5086 | -10,585 | -13,248 | -4,0997 | YOCNT |
| 48 | -3,7037 | -7,9917 | -15,207 | -5,5941 | YOCNT |

**Table S5** LDA confusion matrix for ileum samples in the full (4000-650 cm^-1^) spectral region. YOcnt (aged control rats), OYcnt (young control rats), YOpls (young plasma recipient aged rats), OYpls (aged plasma recipient young rats)

| **Confusion matrix** | **Actual** | **YOCNT** | **YOPLS** | **OYCNT** | **OYPLS** |
| --- | --- | --- | --- | --- | --- |
| **Predicted** |  | **1** | **2** | **3** | **4** |
| **YOCNT** | **1** | **12** | 0 | 1 | 0 |
| **YOPLS** | **2** | 0 | **12** | 1 | 0 |
| **OYCNT** | **3** | 0 | 0 | **10** | 1 |
| **OYPLS** | **4** | 0 | 0 | 0 | **11** |

**Table S6** LDA prediction matrix for ileum samples in the full (4000-650 cm^-1^) spectral region. YOcnt (aged control rats), OYcnt (young control rats), YOpls (young plasma recipient aged rats), OYpls (aged plasma recipient young rats)

| **Prediction**  **matrix** | **YOCNT** | **YOPLS** | **OYCNT** | **OYPLS** | **Predicted** |
| --- | --- | --- | --- | --- | --- |
| Samples | 1 | 2 | 3 | 4 | 5 |
| 1 | -2,32412 | -11,805 | -9,82084 | -28,7604 | YOCNT |
| 2 | -5,47868 | -9,2624 | -12,0441 | -32,621 | YOCNT |
| 3 | -2,22118 | -13,3729 | -10,4939 | -26,1709 | YOCNT |
| 4 | -2,77308 | -9,0595 | -10,3264 | -21,2403 | YOCNT |
| 5 | -2,26783 | -13,1477 | -12,4652 | -33,0736 | YOCNT |
| 6 | -2,57622 | -12,006 | -8,12398 | -24,6256 | YOCNT |
| 7 | -19,7165 | -32,3983 | -30,9832 | -47,8933 | YOCNT |
| 8 | -2,66225 | -10,2039 | -12,4032 | -26,7207 | YOCNT |
| 9 | -5,2901 | -14,3236 | -13,1429 | -34,5561 | YOCNT |
| 10 | -1,78104 | -9,9942 | -9,50991 | -24,2787 | YOCNT |
| 11 | -3,2 | -13,8713 | -9,86227 | -22,1725 | YOCNT |
| 12 | -1,97534 | -9,51717 | -9,4897 | -22,1887 | YOCNT |
| 13 | -32,2224 | -20,4345 | -25,781 | -35,4842 | YOPLS |
| 14 | -19,3463 | -6,07543 | -15,0255 | -27,2215 | YOPLS |
| 15 | -6,07871 | -2,70618 | -3,89587 | -14,4608 | YOPLS |
| 16 | -9,24715 | -2,3581 | -3,09148 | -10,395 | YOPLS |
| 17 | -7,00996 | -2,61182 | -4,72038 | -13,2757 | YOPLS |
| 18 | -9,67844 | -1,87309 | -6,5198 | -18,2284 | YOPLS |
| 19 | -12,8462 | -1,82748 | -6,35874 | -15,1559 | YOPLS |
| 20 | -11,4035 | -1,65831 | -6,56657 | -15,0174 | YOPLS |
| 21 | -10,13 | -2,53309 | -6,3948 | -17,6498 | YOPLS |
| 22 | -15,9997 | -2,27318 | -8,06888 | -15,2114 | YOPLS |
| 23 | -11,1533 | -1,69895 | -5,20406 | -14,6368 | YOPLS |
| 24 | -9,82945 | -2,19946 | -5,62141 | -12,9542 | YOPLS |
| 25 | -13,4789 | -11,4754 | -3,31991 | -16,532 | OYCNT |
| 26 | -9,76783 | -3,73431 | -1,92025 | -10,1329 | OYCNT |
| 27 | -12,979 | -7,4545 | -1,99267 | -9,75743 | OYCNT |
| 28 | -17,0732 | -16,6933 | -8,50266 | -15,911 | OYCNT |
| 29 | -11,8116 | -6,81219 | -4,13095 | -16,6497 | OYCNT |
| 30 | -19,1611 | -13,7007 | -5,86342 | -17,1235 | OYCNT |
| 31 | -12,0045 | -2,50741 | -3,83557 | -9,1528 | YOPLS |
| 32 | -10,6228 | -4,59455 | -3,04588 | -8,97122 | OYCNT |
| 33 | -7,16452 | -4,41249 | -2,70885 | -10,7974 | OYCNT |
| 34 | -20,7051 | -8,39462 | -4,84613 | -14,8934 | OYCNT |
| 35 | -17,6578 | -9,71374 | -4,71256 | -16,0361 | OYCNT |
| 36 | -5,47205 | -21,0049 | -16,6203 | -35,1961 | YOCNT |
| 37 | -17,419 | -9,98383 | -4,97865 | -10,1333 | OYCNT |
| 38 | -18,6503 | -5,69861 | -6,28289 | -4,75181 | OYPLS |
| 39 | -17,7643 | -9,29826 | -7,6938 | -2,66975 | OYPLS |
| 40 | -21,5701 | -10,7429 | -10,1918 | -2,24294 | OYPLS |
| 41 | -24,2049 | -15,7941 | -9,42986 | -2,47619 | OYPLS |
| 42 | -39,1444 | -26,5562 | -21,8034 | -11,4035 | OYPLS |
| 43 | -32,8861 | -26,0025 | -15,7576 | -4,95711 | OYPLS |
| 44 | -30,0061 | -18,0417 | -15,6814 | -2,03346 | OYPLS |
| 45 | -41,1297 | -29,2758 | -21,805 | -4,39633 | OYPLS |
| 46 | -40,2532 | -25,0294 | -19,8367 | -3,54982 | OYPLS |
| 47 | -34,1975 | -23,5131 | -21,4748 | -3,65089 | OYPLS |
| 48 | -33,3369 | -20,0321 | -23,2456 | -6,26197 | OYPLS |

**Table S7** LDA confusion matrix for ileum samples in the spectral region of nucleic acids and polysaccharides (1200-650 cm^-1^). YOcnt (aged control rats), OYcnt (young control rats), YOpls (young plasma recipient aged rats), OYpls (aged plasma recipient young rats)

| **Confusion matrix** | **Actual** | **YOCNT** | **YOPLS** | **OYCNT** | **OYPLS** |
| --- | --- | --- | --- | --- | --- |
| **Predicted** |  | **1** | **2** | **3** | **4** |
| **YOCNT** | **1** | **12** | 0 | 1 | 0 |
| **YOPLS** | **2** | 0 | **12** | 2 | 1 |
| **OYCNT** | **3** | 0 | 0 | **9** | 0 |
| **OYPLS** | **4** | 0 | 0 | 0 | **11** |

**Table S8** LDA prediction matrix for ileum samples in the spectral region of nucleic acids and polysaccharides (1200-650 cm^-1^). YOcnt (aged control rats), OYcnt (young control rats), YOpls (young plasma recipient aged rats), OYpls (aged plasma recipient young rats)

| **Prediction Matrix** | **YOCNT** | **YOPLS** | **OYCNT** | **OYPLS** | **Predicted** |
| --- | --- | --- | --- | --- | --- |
| Sample | 1 | 2 | 3 | 4 | 5 |
| 1 | -2,3409 | -15,3152 | -16,0541 | -8,7110 | YOCNT |
| 2 | -4,3500 | -10,0416 | -14,3054 | -9,7191 | YOCNT |
| 3 | -2,5071 | -19,0794 | -19,6596 | -11,2071 | YOCNT |
| 4 | -2,5896 | -17,0415 | -18,0389 | -10,1898 | YOCNT |
| 5 | -2,6517 | -16,7399 | -18,4252 | -11,7289 | YOCNT |
| 6 | -2,8857 | -14,2964 | -12,8069 | -6,5166 | YOCNT |
| 7 | -20,0469 | -35,7590 | -36,6882 | -29,5627 | YOCNT |
| 8 | -2,4187 | -11,4111 | -14,6298 | -8,9888 | YOCNT |
| 9 | -4,8804 | -11,6484 | -13,8196 | -9,1860 | YOCNT |
| 10 | -1,8442 | -13,1986 | -14,8026 | -8,4187 | YOCNT |
| 11 | -2,6706 | -16,0187 | -14,3120 | -6,9457 | YOCNT |
| 12 | -2,1266 | -9,9455 | -11,5482 | -6,2425 | YOCNT |
| 13 | -33,3577 | -20,8001 | -26,6729 | -30,9615 | YOPLS |
| 14 | -18,2408 | -2,9352 | -8,4695 | -12,3682 | YOPLS |
| 15 | -7,8530 | -2,5497 | -5,0247 | -5,1184 | YOPLS |
| 16 | -10,3811 | -2,3584 | -2,9426 | -4,3012 | YOPLS |
| 17 | -8,4889 | -2,1481 | -4,7037 | -5,4723 | YOPLS |
| 18 | -16,2730 | -1,9981 | -7,2127 | -10,8185 | YOPLS |
| 19 | -16,6537 | -2,0008 | -6,3685 | -10,1632 | YOPLS |
| 20 | -14,0258 | -1,6813 | -6,2176 | -9,1009 | YOPLS |
| 21 | -13,8402 | -2,7374 | -6,8290 | -10,0813 | YOPLS |
| 22 | -20,1138 | -2,6795 | -6,9027 | -12,1451 | YOPLS |
| 23 | -14,1491 | -1,6954 | -4,7596 | -7,8885 | YOPLS |
| 24 | -11,6013 | -2,2116 | -5,5382 | -7,5076 | YOPLS |
| 25 | -19,7459 | -11,1652 | -5,8847 | -7,6218 | OYCNT |
| 26 | -12,1497 | -3,2112 | -3,4256 | -5,0394 | YOPLS |
| 27 | -15,6054 | -7,9844 | -1,9795 | -3,9774 | OYCNT |
| 28 | -20,9006 | -15,8877 | -9,7073 | -9,8168 | OYCNT |
| 29 | -19,0180 | -13,2090 | -10,2030 | -12,6413 | OYCNT |
| 30 | -36,7497 | -20,0309 | -10,4420 | -15,1735 | OYCNT |
| 31 | -12,1827 | -2,0223 | -3,2485 | -5,6568 | YOPLS |
| 32 | -14,8693 | -4,5990 | -3,0333 | -5,2178 | OYCNT |
| 33 | -21,0550 | -7,2639 | -4,1146 | -7,3810 | OYCNT |
| 34 | -27,4245 | -9,0741 | -4,7086 | -10,6377 | OYCNT |
| 35 | -25,3754 | -11,6982 | -5,7619 | -11,3187 | OYCNT |
| 36 | -4,9411 | -15,9395 | -13,7303 | -6,7282 | YOCNT |
| 37 | -12,8076 | -13,2500 | -7,6505 | -7,6241 | OYPLS |
| 38 | -6,4670 | -4,0179 | -3,8824 | -3,0054 | OYPLS |
| 39 | -5,8092 | -5,5264 | -5,1493 | -3,1884 | OYPLS |
| 40 | -5,4867 | -5,3004 | -4,3420 | -2,4159 | OYPLS |
| 41 | -10,2301 | -13,2703 | -5,8366 | -2,6857 | OYPLS |
| 42 | -16,3856 | -21,0476 | -12,3042 | -7,9960 | OYPLS |
| 43 | -13,1740 | -18,8595 | -8,5669 | -4,7637 | OYPLS |
| 44 | -6,8371 | -7,9883 | -4,5483 | -1,7975 | OYPLS |
| 45 | -13,1113 | -14,7569 | -5,7704 | -3,0135 | OYPLS |
| 46 | -13,2072 | -11,1920 | -3,6804 | -2,4214 | OYPLS |
| 47 | -11,5447 | -7,6595 | -4,3705 | -3,1608 | OYPLS |
| 48 | -8,2388 | -4,4572 | -6,0647 | -5,1226 | YOPLS |

**Table S9** SVM classification for ileum samples in the full (4000-650 cm^-1^) spectral region. YOcnt (aged control rats), OYcnt (young control rats), YOpls (young plasma recipient aged rats), OYpls (aged plasma recipient young rats). Classification (nu-SVC). Method: Linear. *Highlights the wrong classification

| **Accuracy (%)** | | | |
| --- | --- | --- | --- |
| Training | 87.50 | Validation | 77.08 |
| **Classification** | | | |
| Samples | | Class | |
| YOcnt | 1 | 1 | YOcnt |
| **YOcnt** | **2** | **2** | ***YOpls** |
| YOcnt | 3 | 3 | YOcnt |
| YOcnt | 4 | 4 | YOcnt |
| YOcnt | 5 | 5 | YOcnt |
| YOcnt | 6 | 6 | YOcnt |
| YOcnt | 7 | 7 | YOcnt |
| YOcnt | 8 | 8 | YOcnt |
| YOcnt | 9 | 9 | YOcnt |
| YOcnt | 10 | 10 | YOcnt |
| YOcnt | 11 | 11 | YOcnt |
| YOcnt | 12 | 12 | YOcnt |
| YOpls | 13 | 13 | YOpls |
| YOpls | 14 | 14 | YOpls |
| **YOpls** | **15** | **15** | ***YOcnt** |
| YOpls | 16 | 16 | YOpls |
| YOpls | 17 | 17 | YOpls |
| YOpls | 18 | 18 | YOpls |
| YOpls | 19 | 19 | YOpls |
| YOpls | 20 | 20 | YOpls |
| YOpls | 21 | 21 | YOpls |
| YOpls | 22 | 22 | YOpls |
| YOpls | 23 | 23 | YOpls |
| YOpls | 24 | 24 | YOpls |
| OYcnt | 25 | 25 | OYcnt |
| OYcnt | 26 | 26 | OYcnt |
| OYcnt | 27 | 27 | OYcnt |
| OYcnt | 28 | 28 | OYcnt |
| OYcnt | 29 | 29 | OYcnt |
| OYcnt | 30 | 30 | OYcnt |
| **OYcnt** | **31** | **31** | ***YOpls** |
| **OYcnt** | **32** | **32** | ***YOcnt** |
| OYcnt | 33 | 33 | OYcnt |
| OYcnt | 34 | 34 | OYcnt |
| OYcnt | 35 | 35 | OYcnt |
| **OYcnt** | **36** | **36** | ***YOcnt** |
| **OYpls** | **37** | **37** | ***OYcnt** |
| OYpls | 38 | 38 | OYpls |
| OYpls | 39 | 39 | OYpls |
| OYpls | 40 | 40 | OYpls |
| OYpls | 41 | 41 | OYpls |
| OYpls | 42 | 42 | OYpls |
| OYpls | 43 | 43 | OYpls |
| OYpls | 44 | 44 | OYpls |
| OYpls | 45 | 45 | OYpls |
| OYpls | 46 | 46 | OYpls |
| OYpls | 47 | 47 | OYpls |
| **OYpls** | **48** | **48** | ***YOpls** |

**Table S10** LDA confusion matrix for colon samples in lipid (3000-2700 cm^-1^) spectral region. YOcnt (aged control rats), OYcnt (young control rats), YOpls (young plasma recipient aged rats), OYpls (aged plasma recipient young rats)

| **Confusion matrix** | **Actual** | **YOCNT** | **YOPLS** | **OYCNT** | **OYPLS** |
| --- | --- | --- | --- | --- | --- |
| **Predicted** |  | **1** | **2** | **3** | **4** |
| **YOCNT** | **1** | **10** | 0 | 3 | 1 |
| **YOPLS** | **2** | 1 | **10** | 1 | 0 |
| **OYCNT** | **3** | 0 | 2 | **8** | 0 |
| **OYPLS** | **4** | 1 | 0 | 0 | **11** |

**Table S11** LDA prediction matrix for colon samples in lipid (3000-2700 cm^-1^) spectral region. YOcnt (aged control rats), OYcnt (young control rats), YOpls (young plasma recipient aged rats), OYpls (aged plasma recipient young rats)

| **Prediction Matrix** | **YOCNT** | **YOPLS** | **OYCNT** | **OYPLS** | **Predicted** |
| --- | --- | --- | --- | --- | --- |
| Sample | 1 | 2 | 3 | 4 | 5 |
| 1 | -4,345 | -12,862 | -8,6041 | -10,893 | YOCNT |
| 2 | -6,0897 | -9,4722 | -7,9416 | -10,723 | YOCNT |
| 3 | -5,9972 | -10,685 | -10,896 | -8,6171 | YOCNT |
| 4 | -11,413 | -8,6831 | -12,591 | -6,2841 | OYPLS |
| 5 | -3,3959 | -3,1047 | -3,6146 | -5,9436 | YOPLS |
| 6 | -15,613 | -18,139 | -16,047 | -28,561 | YOCNT |
| 7 | -2,7755 | -7,6001 | -4,3017 | -8,9986 | YOCNT |
| 8 | -3,6635 | -8,6303 | -5,6806 | -7,4517 | YOCNT |
| 9 | -2,7006 | -6,9739 | -5,1168 | -6,4263 | YOCNT |
| 10 | -1,8659 | -6,0098 | -5,1894 | -5,6873 | YOCNT |
| 11 | -2,7621 | -3,2202 | -2,9096 | -3,7708 | YOCNT |
| 12 | -4,7954 | -7,2387 | -4,9034 | -7,6399 | YOCNT |
| 13 | -5,8217 | -1,9811 | -4,4829 | -4,9015 | YOPLS |
| 14 | -5,5248 | -2,3716 | -2,992 | -5,0293 | YOPLS |
| 15 | -6,4101 | -3,2709 | -4,1145 | -6,2427 | YOPLS |
| 16 | -6,7485 | -3,1344 | -3,698 | -8,1259 | YOPLS |
| 17 | -4,4901 | -2,1936 | -2,4045 | -5,7613 | YOPLS |
| 18 | -13,064 | -11,261 | -9,9399 | -11,614 | OYCNT |
| 19 | -7,418 | -3,478 | -7,1689 | -4,0091 | YOPLS |
| 20 | -7,9115 | -5,5427 | -8,2266 | -7,4234 | YOPLS |
| 21 | -4,7104 | -2,3632 | -3,5911 | -5,3867 | YOPLS |
| 22 | -5,6772 | -3,5713 | -4,2156 | -5,6965 | YOPLS |
| 23 | -2,9547 | -2,4706 | -2,1497 | -5,1915 | OYCNT |
| 24 | -15,397 | -7,2874 | -12,231 | -12,573 | YOPLS |
| 25 | -3,5745 | -7,5708 | -3,891 | -8,2334 | YOCNT |
| 26 | -17,817 | -10,702 | -9,7787 | -15,998 | OYCNT |
| 27 | -14,231 | -13,827 | -10,999 | -17,635 | OYCNT |
| 28 | -5,9905 | -3,3654 | -2,8134 | -8,3177 | OYCNT |
| 29 | -6,0601 | -3,566 | -4,1147 | -7,4748 | YOPLS |
| 30 | -11,488 | -11,665 | -9,5925 | -17,788 | OYCNT |
| 31 | -2,5765 | -2,3606 | -1,9166 | -3,9665 | OYCNT |
| 32 | -2,1224 | -5,1685 | -2,8546 | -6,5948 | YOCNT |
| 33 | -5,5009 | -6,3868 | -3,3793 | -8,8748 | OYCNT |
| 34 | -5,9309 | -3,8141 | -3,717 | -6,0841 | OYCNT |
| 35 | -3,0718 | -3,5945 | -3,1124 | -4,4161 | YOCNT |
| 36 | -2,6231 | -2,8761 | -2,4393 | -4,0132 | OYCNT |
| 37 | -9,7976 | -5,457 | -6,7353 | -4,5832 | OYPLS |
| 38 | -8,1959 | -7,0821 | -6,9251 | -3,4427 | OYPLS |
| 39 | -7,2945 | -5,712 | -7,0322 | -2,3673 | OYPLS |
| 40 | -9,61 | -6,2991 | -9,0716 | -3,052 | OYPLS |
| 41 | -9,9911 | -6,1731 | -7,5109 | -4,59 | OYPLS |
| 42 | -7,1047 | -4,9132 | -6,2586 | -2,3024 | OYPLS |
| 43 | -7,1214 | -7,738 | -7,2805 | -3,8102 | OYPLS |
| 44 | -6,4564 | -7,3786 | -8,9567 | -2,7116 | OYPLS |
| 45 | -9,7708 | -10,961 | -13,385 | -4,239 | OYPLS |
| 46 | -5,5531 | -7,5782 | -9,9813 | -4,4259 | OYPLS |
| 47 | -8,7305 | -7,2329 | -10,375 | -6,6428 | OYPLS |
| 48 | -3,5449 | -4,0958 | -4,8667 | -5,4244 | YOCNT |

**Table S12** LDA confusion matrix for colon samples in protein (1700-1500 cm^-1^) spectral region. YOcnt (aged control rats), OYcnt (young control rats), YOpls (young plasma recipient aged rats), OYpls (aged plasma recipient young rats)

| **Confusion matrix** | **Actual** | **YOCNT** | **YOPLS** | **OYCNT** | **OYPLS** |
| --- | --- | --- | --- | --- | --- |
| **Predicted** |  | **1** | **2** | **3** | **4** |
| **YOCNT** | **1** | **12** | 0 | 0 | 1 |
| **YOPLS** | **2** | 0 | **11** | 1 | 2 |
| **OYCNT** | **3** | 0 | 1 | **11** | 2 |
| **OYPLS** | **4** | 0 | 0 | 0 | **7** |

**Table S13** LDA prediction matrix for colon samples in protein (1700-1500 cm^-1^) spectral region. YOcnt (aged control rats), OYcnt (young control rats), YOpls (young plasma recipient aged rats), OYpls (aged plasma recipient young rats)

| **Prediction Matrix** | **YOCNT** | **YOPLS** | **OYCNT** | **OYPLS** | **Predicted** |
| --- | --- | --- | --- | --- | --- |
| Sample | 1 | 2 | 3 | 4 | 5 |
| 1 | -1,9337 | -9,9374 | -9,2 | -9,2101 | YOCNT |
| 2 | -3,3133 | -9,2885 | -9,6363 | -10,329 | YOCNT |
| 3 | -9,0462 | -12,125 | -16,282 | -16,676 | YOCNT |
| 4 | -8,1095 | -13,827 | -15,866 | -11,037 | YOCNT |
| 5 | -3,7158 | -10,087 | -11,414 | -8,9496 | YOCNT |
| 6 | -7,4332 | -16,413 | -19,124 | -16,656 | YOCNT |
| 7 | -2,6025 | -4,3058 | -5,2753 | -5,6747 | YOCNT |
| 8 | -3,0757 | -12,855 | -10,006 | -10,721 | YOCNT |
| 9 | -3,4672 | -7,379 | -6,0208 | -5,5143 | YOCNT |
| 10 | -3,1936 | -12,358 | -13,036 | -12,542 | YOCNT |
| 11 | -7,4594 | -13,278 | -13,208 | -18,509 | YOCNT |
| 12 | -7,0811 | -10,133 | -8,2264 | -9,6806 | YOCNT |
| 13 | -7,5349 | -3,6553 | -5,6046 | -4,7999 | YOPLS |
| 14 | -8,3812 | -2,8878 | -3,6887 | -4,2072 | YOPLS |
| 15 | -12,438 | -4,4573 | -6,4072 | -6,7356 | YOPLS |
| 16 | -8,3638 | -2,9707 | -4,3191 | -5,8091 | YOPLS |
| 17 | -9,3787 | -3,2877 | -5,5171 | -5,6623 | YOPLS |
| 18 | -8,8851 | -8,7117 | -5,5661 | -8,5262 | OYCNT |
| 19 | -7,1167 | -5,2059 | -10,576 | -10,975 | YOPLS |
| 20 | -14,514 | -6,0139 | -11,754 | -13,308 | YOPLS |
| 21 | -17,742 | -7,347 | -10,186 | -7,4155 | YOPLS |
| 22 | -9,2892 | -3,7008 | -3,7522 | -3,8404 | YOPLS |
| 23 | -9,1437 | -2,7507 | -4,0229 | -4,4894 | YOPLS |
| 24 | -15,372 | -5,6138 | -12,136 | -12,693 | YOPLS |
| 25 | -12,102 | -9,9488 | -3,8931 | -5,554 | OYCNT |
| 26 | -16,754 | -6,2004 | -4,5276 | -7,4866 | OYCNT |
| 27 | -13,928 | -7,0822 | -3,0268 | -5,1577 | OYCNT |
| 28 | -14,294 | -7,748 | -3,8503 | -7,4827 | OYCNT |
| 29 | -9,3767 | -4,6382 | -4,1746 | -4,5686 | OYCNT |
| 30 | -8,1169 | -6,6759 | -4,5898 | -7,9945 | OYCNT |
| 31 | -7,1098 | -2,4244 | -3,6024 | -2,5096 | YOPLS |
| 32 | -5,3239 | -4,0078 | -1,8046 | -3,5101 | OYCNT |
| 33 | -12,992 | -7,7092 | -3,4954 | -6,5836 | OYCNT |
| 34 | -13,166 | -4,7114 | -4,484 | -5,2592 | OYCNT |
| 35 | -6,404 | -8,1642 | -5,9283 | -6,5542 | OYCNT |
| 36 | -4,4667 | -4,7884 | -3,7944 | -4,7414 | OYCNT |
| 37 | -13,435 | -5,1627 | -7,1901 | -5,6511 | YOPLS |
| 38 | -11,861 | -7,2466 | -4,8565 | -3,9119 | OYPLS |
| 39 | -11,441 | -4,763 | -3,8532 | -2,9813 | OYPLS |
| 40 | -9,8983 | -3,2376 | -4,1783 | -3,2399 | YOPLS |
| 41 | -9,7716 | -3,7735 | -3,5737 | -4,0432 | OYCNT |
| 42 | -12,138 | -4,6851 | -2,9825 | -2,5687 | OYPLS |
| 43 | -13,375 | -8,935 | -4,5555 | -4,5678 | OYCNT |
| 44 | -7,2428 | -5,9024 | -5,4889 | -3,4329 | OYPLS |
| 45 | -3,8966 | -8,8039 | -6,9364 | -5,0031 | YOCNT |
| 46 | -4,5492 | -6,5295 | -6,5303 | -3,2858 | OYPLS |
| 47 | -25,625 | -19,848 | -18,383 | -11,785 | OYPLS |
| 48 | -8,1702 | -9,3088 | -8,0391 | -5,8664 | OYPLS |

**Table S14** LDA confusion matrix for colon samples in the full (4000-650 cm^-1^) spectral region. YOcnt (aged control rats), OYcnt (young control rats), YOpls (young plasma recipient aged rats), OYpls (aged plasma recipient young rats)

| **Confusion matrix** | **Actual** | **YOCNT** | **YOPLS** | **OYCNT** | **OYPLS** |
| --- | --- | --- | --- | --- | --- |
| **Predicted** |  | **1** | **2** | **3** | **4** |
| **YOCNT** | **1** | **10** | 0 | 0 | 0 |
| **YOPLS** | **2** | 0 | **10** | 1 | 2 |
| **OYCNT** | **3** | 0 | 0 | **9** | 2 |
| **OYPLS** | **4** | 2 | 2 | 2 | **8** |

**Table S15** LDA prediction matrix for colon samples in the full (4000-650 cm^-1^) spectral region. YOcnt (aged control rats), OYcnt (young control rats), YOpls (young plasma recipient aged rats), OYpls (aged plasma recipient young rats)

| **Prediction matrix** | **YOCNT** | **YOPLS** | **OYCNT** | **OYPLS** | **Predicted** |
| --- | --- | --- | --- | --- | --- |
| Samples | 1 | 2 | 3 | 4 | 5 |
| 1 | -3,08296 | -9,84624 | -9,15757 | -4,71659 | YOCNT |
| 2 | -3,8902 | -7,71364 | -12,0942 | -6,22727 | YOCNT |
| 3 | -6,00032 | -8,61711 | -16,0393 | -8,85274 | YOCNT |
| 4 | -7,37768 | -10,8724 | -17,8132 | -9,30306 | YOCNT |
| 5 | -5,6129 | -11,7551 | -14,467 | -8,47085 | YOCNT |
| 6 | -12,5435 | -21,0408 | -22,3637 | -17,2519 | YOCNT |
| 7 | -3,42547 | -4,11612 | -3,77172 | -2,25231 | OYPLS |
| 8 | -4,42168 | -11,8534 | -12,1969 | -6,85767 | YOCNT |
| 9 | -2,82693 | -6,6456 | -7,77483 | -3,45577 | YOCNT |
| 10 | -3,0981 | -8,34204 | -9,13963 | -4,88203 | YOCNT |
| 11 | -3,29386 | -3,46302 | -5,63206 | -3,45736 | YOCNT |
| 12 | -5,19871 | -5,88496 | -4,90287 | -3,45103 | OYPLS |
| 13 | -4,11786 | -2,2456 | -7,77318 | -4,43529 | YOPLS |
| 14 | -6,5043 | -3,22106 | -6,26294 | -5,53676 | YOPLS |
| 15 | -9,88707 | -5,243 | -8,41696 | -9,19501 | YOPLS |
| 16 | -8,0067 | -3,06865 | -6,20698 | -6,15661 | YOPLS |
| 17 | -6,92221 | -2,86348 | -5,95297 | -5,92874 | YOPLS |
| 18 | -14,5514 | -13,9941 | -15,5825 | -12,6004 | OYPLS |
| 19 | -7,18818 | -3,25885 | -10,8733 | -7,5774 | YOPLS |
| 20 | -10,5294 | -4,46431 | -10,7438 | -9,39495 | YOPLS |
| 21 | -6,94657 | -2,15731 | -5,65943 | -5,02432 | YOPLS |
| 22 | -6,70184 | -3,88464 | -3,75201 | -3,52964 | OYPLS |
| 23 | -6,40568 | -2,42724 | -4,25311 | -4,76547 | YOPLS |
| 24 | -13,4219 | -4,97662 | -13,8505 | -12,7144 | YOPLS |
| 25 | -6,28417 | -8,87615 | -3,80512 | -4,21921 | OYCNT |
| 26 | -21,6485 | -15,9598 | -11,3146 | -17,112 | OYCNT |
| 27 | -28,5221 | -20,0734 | -12,3792 | -17,9781 | OYCNT |
| 28 | -16,7994 | -11,5388 | -4,21719 | -9,33211 | OYCNT |
| 29 | -7,94817 | -5,66672 | -4,14651 | -4,18758 | OYCNT |
| 30 | -5,59918 | -5,46123 | -2,55752 | -3,02412 | OYCNT |
| 31 | -4,11034 | -2,07986 | -3,80828 | -3,02662 | YOPLS |
| 32 | -5,94378 | -6,17021 | -2,33777 | -3,30238 | OYCNT |
| 33 | -14,236 | -11,9481 | -4,43485 | -9,55466 | OYCNT |
| 34 | -10,2698 | -6,59608 | -3,56477 | -7,09182 | OYCNT |
| 35 | -6,26574 | -6,76367 | -3,44054 | -3,00196 | OYPLS |
| 36 | -5,45622 | -4,89142 | -2,49634 | -2,47646 | OYPLS |
| 37 | -8,1871 | -4,41943 | -9,92713 | -7,57488 | YOPLS |
| 38 | -4,31903 | -4,68339 | -4,96143 | -3,24572 | OYPLS |
| 39 | -3,55605 | -2,91307 | -4,30437 | -2,24853 | OYPLS |
| 40 | -4,94498 | -2,61933 | -5,08202 | -3,15269 | YOPLS |
| 41 | -4,76095 | -3,23533 | -3,25524 | -3,06987 | OYPLS |
| 42 | -5,01401 | -3,98667 | -1,88303 | -2,08023 | OYCNT |
| 43 | -8,32911 | -9,74958 | -3,89269 | -4,8626 | OYCNT |
| 44 | -3,12925 | -6,0376 | -5,10901 | -2,6503 | OYPLS |
| 45 | -3,56533 | -9,55454 | -8,15813 | -3,45277 | OYPLS |
| 46 | -4,06046 | -9,47904 | -8,5804 | -4,00308 | OYPLS |
| 47 | -9,32891 | -15,517 | -11,7113 | -7,07029 | OYPLS |
| 48 | -8,67339 | -12,3215 | -8,40198 | -6,0514 | OYPLS |

**Table S16** LDA confusion matrix for colon samples in the spectral region of nucleic acids and polysaccharides (1200-650 cm^-1^). YOcnt (aged control rats), OYcnt (young control rats), YOpls (young plasma recipient aged rats), OYpls (aged plasma recipient young rats)

| **Confusion matrix** | **Actual** | **YOCNT** | **YOPLS** | **OYCNT** | **OYPLS** |
| --- | --- | --- | --- | --- | --- |
| **Predicted** |  | **1** | **2** | **3** | **4** |
| **YOCNT** | **1** | **9** | 0 | 0 | 3 |
| **YOPLS** | **2** | 0 | **11** | 2 | 1 |
| **OYCNT** | **3** | 1 | 0 | **10** | 0 |
| **OYPLS** | **4** | 2 | 1 | 0 | **8** |

**Table S17** LDA prediction matrix for colon samples in the spectral region of nucleic acids and polysaccharides (1200-650 cm^-1^). YOcnt (aged control rats), OYcnt (young control rats), YOpls (young plasma recipient aged rats), OYpls (aged plasma recipient young rats)

| **Prediction Matrix** | **YOCNT** | **YOPLS** | **OYCNT** | **OYPLS** | **Predicted** |
| --- | --- | --- | --- | --- | --- |
| Sample | 1 | 2 | 3 | 4 | 5 |
| 1 | -2,9542 | -10,815 | -11,314 | -5,4121 | YOCNT |
| 2 | -3,8667 | -7,1961 | -9,9627 | -4,1558 | YOCNT |
| 3 | -4,9406 | -8,657 | -18,007 | -6,4788 | YOCNT |
| 4 | -5,8138 | -11,089 | -18,159 | -5,8482 | YOCNT |
| 5 | -7,1047 | -12,33 | -13,749 | -8,9665 | YOCNT |
| 6 | -9,6131 | -18,457 | -19,897 | -16,317 | YOCNT |
| 7 | -4,308 | -4,7403 | -3,885 | -4,5519 | OYCNT |
| 8 | -4,6488 | -12,982 | -16,961 | -6,0136 | YOCNT |
| 9 | -4,1795 | -8,7313 | -15,773 | -4,3095 | YOCNT |
| 10 | -4,1077 | -10,652 | -13,061 | -6,558 | YOCNT |
| 11 | -3,4998 | -4,1059 | -7,0109 | -3,2884 | OYPLS |
| 12 | -4,6563 | -5,2857 | -8,0896 | -3,5333 | OYPLS |
| 13 | -4,2014 | -3,7543 | -10,337 | -3,933 | YOPLS |
| 14 | -7,0081 | -4,0867 | -8,3176 | -5,0256 | YOPLS |
| 15 | -12,59 | -6,0361 | -15,546 | -9,6738 | YOPLS |
| 16 | -8,4979 | -3,1789 | -6,8889 | -6,3228 | YOPLS |
| 17 | -8,7838 | -3,0523 | -5,9843 | -7,0691 | YOPLS |
| 18 | -13,925 | -14,798 | -17,758 | -9,2254 | OYPLS |
| 19 | -6,5708 | -2,6164 | -8,7911 | -5,8147 | YOPLS |
| 20 | -9,207 | -3,3709 | -11,425 | -8,2273 | YOPLS |
| 21 | -9,1845 | -2,405 | -9,0931 | -7,4811 | YOPLS |
| 22 | -9,358 | -4,508 | -4,7102 | -7,5839 | YOPLS |
| 23 | -8,4054 | -3,1503 | -5,524 | -8,2914 | YOPLS |
| 24 | -12,742 | -4,1694 | -12,311 | -10,996 | YOPLS |
| 25 | -4,9812 | -7,0014 | -3,8954 | -5,1156 | OYCNT |
| 26 | -18,528 | -11,382 | -4,2009 | -15,82 | OYCNT |
| 27 | -29,293 | -23,783 | -14,497 | -28,472 | OYCNT |
| 28 | -18,536 | -14,553 | -5,5929 | -18,112 | OYCNT |
| 29 | -8,9674 | -4,9669 | -5,6718 | -5,9692 | YOPLS |
| 30 | -7,1868 | -6,8616 | -3,2508 | -7,2043 | OYCNT |
| 31 | -5,3622 | -2,3183 | -4,4854 | -4,8783 | YOPLS |
| 32 | -9,8384 | -8,8449 | -2,6483 | -11,32 | OYCNT |
| 33 | -15,518 | -11,565 | -4,6037 | -14,405 | OYCNT |
| 34 | -14,249 | -9,4393 | -3,3494 | -13,568 | OYCNT |
| 35 | -12,437 | -11,568 | -4,6796 | -12,592 | OYCNT |
| 36 | -11,266 | -9,2643 | -3,1124 | -11,459 | OYCNT |
| 37 | -10,791 | -6,2902 | -15,527 | -5,9478 | OYPLS |
| 38 | -4,5534 | -5,9178 | -11,564 | -2,3973 | OYPLS |
| 39 | -5,011 | -4,3034 | -13,269 | -3,0242 | OYPLS |
| 40 | -5,6477 | -3,1362 | -11,27 | -3,4136 | YOPLS |
| 41 | -6,7884 | -4,6673 | -9,2824 | -3,5322 | OYPLS |
| 42 | -4,4789 | -3,192 | -5,7601 | -2,2708 | OYPLS |
| 43 | -6,3432 | -8,5232 | -6,4542 | -5,0163 | OYPLS |
| 44 | -2,463 | -5,6001 | -9,1882 | -2,4351 | OYPLS |
| 45 | -2,8209 | -9,4916 | -11,259 | -3,0355 | YOCNT |
| 46 | -1,8542 | -7,6461 | -13,174 | -2,9192 | YOCNT |
| 47 | -7,4402 | -13,512 | -14,47 | -7,3076 | OYPLS |
| 48 | -3,2844 | -7,9749 | -13,446 | -4,4362 | YOCNT |

**Table S18** SVM classification for colon samples in the full (4000-650 cm^-1^) spectral region. YOcnt (aged control rats), OYcnt (young control rats), YOpls (young plasma recipient aged rats), OYpls (aged plasma recipient young rats). Classification (nu-SVC). Method: Linear. *Highlights the wrong classification

| **Accuracy (%)** | | | |
| --- | --- | --- | --- |
| Training | 85.42 | Validation | 54.16 |
| **Classification** | | | |
| Samples | | Class | |
| YOcnt | 1 | 1 | YOcnt |
| YOcnt | 2 | 2 | YOcnt |
| YOcnt | 3 | 3 | YOcnt |
| YOcnt | 4 | 4 | YOcnt |
| YOcnt | 5 | 5 | YOcnt |
| YOcnt | 6 | 6 | YOcnt |
| **YOcnt** | **7** | **7** | ***OYpls** |
| YOcnt | 8 | 8 | YOcnt |
| YOcnt | 9 | 9 | YOcnt |
| YOcnt | 10 | 10 | YOcnt |
| **YOcnt** | **11** | **11** | ***OYcnt** |
| **YOcnt** | **12** | **12** | ***OYcnt** |
| YOpls | 13 | 13 | YOpls |
| YOpls | 14 | 14 | YOpls |
| YOpls | 15 | 15 | YOpls |
| YOpls | 16 | 16 | YOpls |
| YOpls | 17 | 17 | YOpls |
| **YOpls** | **18** | **18** | ***OYpls** |
| YOpls | 19 | 19 | YOpls |
| YOpls | 20 | 20 | YOpls |
| YOpls | 21 | 21 | YOpls |
| YOpls | 22 | 22 | YOpls |
| YOpls | 23 | 23 | YOpls |
| YOpls | 24 | 24 | YOpls |
| OYcnt | 25 | 25 | OYcnt |
| OYcnt | 26 | 26 | OYcnt |
| OYcnt | 27 | 27 | OYcnt |
| OYcnt | 28 | 28 | OYcnt |
| OYcnt | 29 | 29 | OYcnt |
| OYcnt | 30 | 30 | OYcnt |
| **OYcnt** | **31** | **31** | ***YOpls** |
| OYcnt | 32 | 32 | OYcnt |
| OYcnt | 33 | 33 | OYcnt |
| OYcnt | 34 | 34 | OYcnt |
| OYcnt | 35 | 35 | OYcnt |
| OYcnt | 36 | 36 | OYcnt |
| **OYpls** | **37** | **37** | ***YOpls** |
| OYpls | 38 | 38 | OYpls |
| OYpls | 39 | 39 | OYpls |
| **OYpls** | **40** | **40** | ***YOpls** |
| OYpls | 41 | 41 | OYpls |
| OYpls | 42 | 42 | OYpls |
| OYpls | 43 | 43 | OYpls |
| OYpls | 44 | 44 | OYpls |
| OYpls | 45 | 45 | OYpls |
| OYpls | 46 | 46 | OYpls |
| OYpls | 47 | 47 | OYpls |
| OYpls | 48 | 48 | OYpls |
